# Spatiotemporal Regulation of STING Activity by Linear Ubiquitination Governs Antiviral Immunity

**DOI:** 10.1002/advs.202417660

**Published:** 2025-06-19

**Authors:** Yong Zhang, Yesheng Fu, Lihua Qiang, Mengyuan Zhao, Zhe Lu, Zhuo Zhao, Guoping Chen, Zehui Lei, Qiyao Chai, Pupu Ge, Bingxi Li, Jing Wang, Cui Hua Liu, Lingqiang Zhang

**Affiliations:** ^1^ School of Medicine Tsinghua University Beijing 100084 China; ^2^ Laboratory of Pathogen Microbiology and Immunology Institute of Microbiology Chinese Academy of Sciences Beijing 100101 China; ^3^ State Key Laboratory of Medical Proteomics Beijing Proteome Research Center National Center for Protein Sciences (Beijing) Beijing Institute of Lifeomics Beijing 102206 China; ^4^ Medical School University of Chinese Academy of Sciences Beijing 101408 China

**Keywords:** antiviral immunity, HOIP, linear ubiquitination, OTULIN, STING

## Abstract

The cyclic GMP‐AMP synthase (cGAS)‐stimulator of interferon gene protein (STING) signaling plays a critical role in innate immunity and must be tightly regulated to maintain immune homeostasis, but the mechanism underlying the spatiotemporal regulation of this pathway remains largely elusive. Here, it is shown that during DNA viral infection, the linear ubiquitin chain assembly complex (LUBAC) and ovarian tumor deubiquitinase with linear linkage specificity (OTULIN) reversibly catalyze the linear ubiquitination of STING. At the early stage of the infection, LUBAC promotes STING linear ubiquitination to drive its trafficking from the endoplasmic reticulum (ER) to the Golgi apparatus through binding to the Sec24b subunit of the coat protein complex II (COPII) complex. Later on, OTULIN is recruited to TANK1 binding kinase 1 (TBK1)‐phosphorylated STING and removes its linear ubiquitin chains, thus preventing excessive antiviral immune responses. Together, the study uncovers a linear ubiquitination‐governed spatiotemporal regulatory mechanism that fine‐tunes STING‐driven antiviral immunity.

## Graphical abstract

This study uncovers a spatiotemporal regulation of STING activity by linear ubiquitination in antiviral immunity. At the early stage of the infection, LUBAC promotes STING linear ubiquitination to drive its trafficking from the ER to the Golgi apparatus for activation. OTULIN is then recruited to TBK1‐phosphorylated STING and removes its linear ubiquitin chains, thus preventing excessive antiviral immune responses.

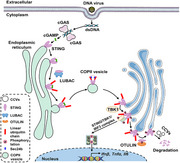

## 1 Introduction

A fine balance between immune activation and suppression is crucial for the effective and appropriate infection control, during which process the immune system is dynamically regulated to ensure optimal host protective immunity while preventing excessive host immune response‐induced immunopathology. The aberrant modulation of this process would lead to various immunological disorders including immune deficiency and autoimmune diseases.^[^ ^1^ ^]^ During infection, certain important molecules and their associated signaling cascades are involved in triggering a prompt activation of innate immunity while maintaining immune homeostasis in a dynamic and coordinated manner, and loss of positive regulators could lead to prolonged infection and pathogen persistence, whereas deficiency of negative factors would promote excessive and persistent pathological damage.^[^ ^2^, ^3^ ^]^ However, the bidirectional regulatory mechanisms involved in controlling the magnitude of the immune responses remain elusive.

The cyclic GMP‐AMP synthase (cGAS)‐stimulator of interferon genes (STING) pathway is an evolutionarily conserved immune signaling pathway, which is critical for detecting a variety of microbial pathogens, especially DNA viruses.^[^ ^4^ ^]^ As an endoplasmic reticulum (ER)‐localized adaptor, STING is highly mobile in cells and its intracellular trafficking‐associated activation needs to be tightly regulated. Upon infection, STING is transported via coat protein complex II (COPII)‐coated vesicles that transport cargos from the ER to ER‐Golgi intermediate compartment (ERGIC) and then to the Golgi,^[^ ^5^, ^6^ ^]^ where it facilitates the recruitment and activation of TANK binding kinase (TBK1) and interferon regulatory factor 3 (IRF3) to induce interferon (IFN) expression.^[^ ^7^ ^]^ Subsequently, STING translocates from the Golgi to post‐Golgi vesicles and then to recycling endosomes (REs), finally reaching lysosomes^[^ ^8^ ^]^ and leading to its rapid degradation and dampening of interferon signaling.^[^ ^6^ ^]^ Previous studies have shown that STING trafficking is a highly regulated process guided by several transport‐related proteins. For instance, a study showed that STING ER exit protein (STEEP) promotes the production of phosphatidylinositol‐3‐phosphate and ER membrane curvature formation, thus inducing COPII‐mediated ER‐to‐Golgi trafficking of STING.^[^ ^9^ ^]^ Another study reported that stromal interaction molecule 1 (STIM1) acts as an ER‐retention factor that anchors STING on the ER and limits STING signaling.^[^ ^10^ ^]^ Besides, STEEP and inactive rhomboid‐like protein 2 (iRhom2), transmembrane emp24 protein transport domain containing 2 (TMED2), Yip1 domain family 5 (YIPF5) have also been shown to play critical roles in mediating STING trafficking from the ER to the Golgi apparatus.^[^ ^11^, ^12^, ^13^ ^]^ However, the dynamic spatiotemporal regulatory mechanisms governing STING trafficking remain largely undefined.

As an atypical ubiquitination, linear ubiquitination (also known as Met1‐linked ubiquitination) is involved in the dynamic and reversible regulation of various immune signaling pathways such as NF‐κB signaling pathway,^[^ ^14^ ^]^ which could be specifically catalyzed by the linear ubiquitin chain assembly complex (LUBAC) and removed by ovarian tumor (OTU) deubiquitinase with linear linkage specificity (OTULIN).^[^ ^15^, ^16^, ^17^ ^]^ LUBAC is composed of three components including the HOIL‐1L‐interacting protein (HOIP, which is the major catalytic core of LUBAC and essential for linear ubiquitin chain formation), heme‐oxidized IRP2 ubiquitin ligase‐1L (HOIL‐1L), and Shank‐associated RH domain interactor (SHARPIN) subunits.^[^ ^18^ ^]^ Linear ubiquitin chains play critical homeostasis regulatory functions as demonstrated by the exhibition of embryonic lethality in *Hoip‐* or *Otulin*‐deficient mice,^[^ ^19^, ^20^, ^21^ ^]^ and dysregulation of the Met1‐Ub machinery is associated with various diseases.^[^ ^22^, ^23^, ^24^, ^25^ ^]^ Furthermore, several studies have reported that linear ubiquitination is implicated in multiple biological processes, such as NF‐κB essential modulator (NEMO)‐mediated NF‐κB signaling,^[^ ^26^ ^]^ receptor interacting serine/threonine kinase 1 (RIPK1)‐related cell death,^[^ ^27^ ^]^ apoptosis‐associated speck‐like protein containing a CARD (ASC)‐dependent inflammasome activation,^[^ ^28^ ^]^ autophagy related gene 13 (ATG13)‐associated autophagy and activin receptor‐like kinase 1 (ALK1)‐mediated angiogenesis,^[^ ^29^, ^30^ ^]^ Hypoxia‐inducible factor 1 (HIFɑ)‐dependent lung tumorigenesis,^[^ ^31^ ^]^ Signal transducer and activator of transcription 3 (STAT3)‐associated glioblastoma^[^ ^32^ ^]^ and Centromere‐associated protein E (CENP‐E)‐related chromosome alignment.^[^ ^33^ ^]^ However, the molecular mechanism underlying LUBAC and OTULIN‐governed host immunity against infectious diseases remains largely unknown.

Here, we show that HOIP and OTULIN successively and mutually regulate the linear ubiquitination of STING to fine‐tune cGAS‐STING signaling during different stages of DNA virus infection. HOIP deficiency attenuated anti‐DNA virus immune responses, while OTULIN knockout boosted DNA virus‐triggered immune responses. Moreover, we identified STING as a physiological substrate of linear ubiquitination during DNA virus infection. HOIP interacted with STING and promoted the linear ubiquitination on lysine 338 of STING. Linear ubiquitination of STING facilitated itself transport from ER to Golgi apparatus through interacting with Sec24b subunit of COPII complex. By contrast, OTULIN was recruited by phosphorylated STING to remove the linear ubiquitin chains from STING at the late stage of DNA virus infection, thus avoiding excessive innate immune responses. Our study not only reveals a previously unknown coordinated bidirectional regulatory mechanism of cGAS‐STING signaling pathway by linear ubiquitination, but also provides a potential therapeutic target for STING‐associated infectious and immunological diseases.

## 2 Results

### 2.1 HOIP and OTULIN Bidirectionally Regulate Antiviral Immune Responses Against DNA Viruses

**Figure 1:**
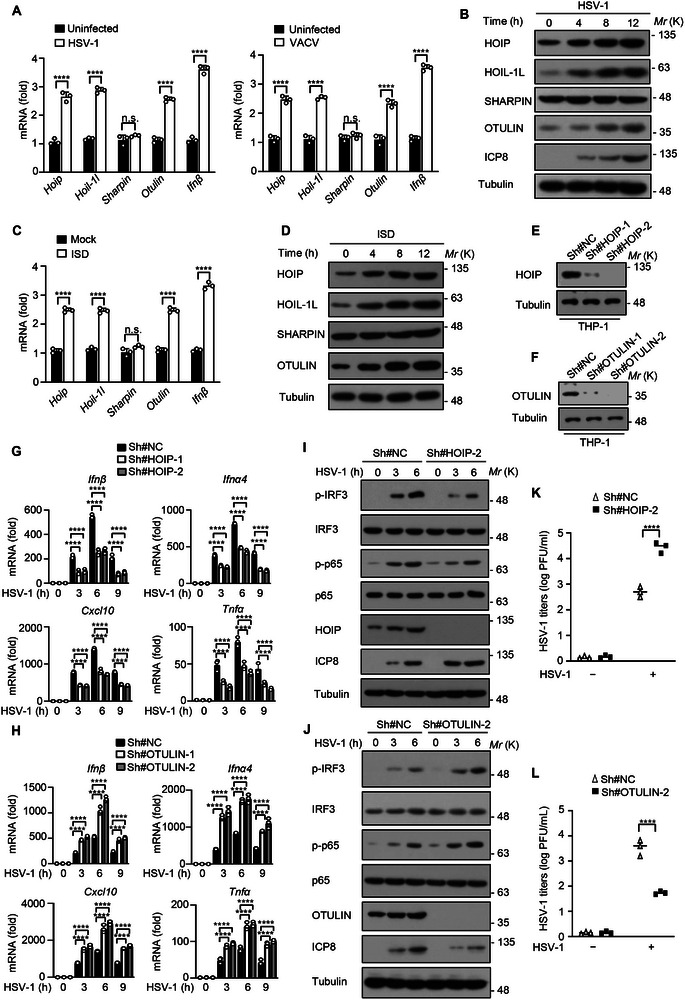
HOIP and OTULIN bidirectionally regulate antiviral immune responses against DNA viruses. A) qRT‐PCR analysis of *Hoip*, *Hoil‐1l, Sharpin*, *Otulin*, or *Ifnβ* mRNA in THP‐1 cells infected with HSV‐1 or VACV for 6 h. B) Immunoblot analysis of HOIP, HOIL‐1L, SHARPIN, OTULIN, ICP8, or Tubulin proteins in THP‐1 cells infected with HSV‐1 for the indicated time points. C) qRT‐PCR analysis of *Hoip*, *Hoil‐1l, Sharpin*, *Otulin*, or *Ifnβ* mRNA in THP‐1 cells treated with 2 µg/mL ISD for 4 h. D) Immunoblot analysis of the HOIP, HOIL‐1L, SHARPIN, OTULIN, or Tubulin proteins in THP‐1 cells stimulated with 2 µg/mL ISD for the indicated time points. E and F) Immunoblot analysis of THP‐1 cells infected with lentivirus expressing scrambled shRNA or shRNA targeting HOIP (E) or OTULIN (F). G and H) qRT‐PCR analysis of *Ifnβ*, *Ifnα4*, *Cxcl10*, or *Tnfα* mRNA in WT, *HOIP* knockdown (G) or *OTULIN* knockdown (H) THP‐1 cells infected with HSV‐1 for the indicated time points. I,J) Immunoblot analysis of p‐IRF3, IRF3, p‐p65, p65, ICP8, Tubulin, HOIP, or OTULIN proteins in WT, *HOIP* knockdown (I) or *OTULIN* knockdown (J) THP‐1 cells infected with HSV‐1 for the indicated time points. K and L) Plaque assay of HSV‐1 titers in WT, *HOIP* knockdown (K) or *OTULIN* knockdown (L) THP‐1 cells infected with HSV‐1 for 6 h. Data are presented as the mean ± SD. Statistical significance was determined by two‐way ANOVA with Sidak's multiple comparisons test (A, C, K, and L) and with Tukey's multiple comparisons test (G and H). ****P < 0.0001; n.s., not significant. Data are representative of three independent experiments.

To explore the potential roles of LUBAC and OTULIN in virus infection, several RNA viruses including vesicular stomatitis virus (VSV) and Sendai virus (SeV), as well as DNA viruses including vaccinia virus (VACV) and herpes simplex virus type 1 (HSV‐1), were used to infect human monocytic leukemia THP‐1 cells, followed by the detection of transcript levels of *Hoip*, *Hoil‐1l*, *Sharpin*, *Otulin*. Our results showed that HSV‐1 and VACV infection induced the upregulation of *Hoip*, *Hoil‐1l*, and *Otulin* mRNA expression (**Figure** **1**A). Although the increase of *Ifnβ* expression was observed upon VSV and SeV infection, no significant change in the transcription of *Hoip*, *Hoil‐1l*, and *Otulin* genes was found in THP‐1 cells infected with SeV and VSV (Figure S1A, Supporting Information). Immunoblot analysis also showed increased expression levels of HOIP, HOIL‐1L, and OTULIN proteins during HSV‐1 infection (Figure 1B). Similar results were observed when THP‐1 cells were transfected with DNA mimic interferon‐stimulatory DNA (ISD), but not RNA mimic poly (I:C) (Figure 1C,D; Figure S1B, Supporting Information), indicating that HOIP and OTULIN are upregulated in response to DNA virus infection, but not RNA virus infection. It was reported that viral infection induces NF‐κB activation and this activation promotes the mRNA level of *HOIP*.^[^ ^34^ ^]^ Consistently, we found that NF‐κB inhibitor PDTC inhibited *Hoip* and *Otulin* mRNA upregulation during DNA virus infection (Figure S1C, Supporting Information). Meanwhile, PDTC showed no effects on *Hoip* and *Otulin* mRNA levels during RNA virus infection (Figure S1D, Supporting Information).

To examine the functions of HOIP and OTULIN in anti‐DNA virus infection, *HOIP* or *OTULIN* knockdown THP‐1 cells were generated via lentivirus transfection and subjected to HSV‐1 virus infection (Figure 1E,F). In *HOIP* knockdown THP‐1 cells, HSV‐1 infection resulted in lower expression of *Ifnβ*, *Ifnα4*, *Cxcl10*, *Tnfα*, and *Il6* at mRNA levels (Figure 1G; Figure S1G, Supporting Information). In contrast, *OTULIN* knockdown led to an increase in the mRNA levels of *Ifnβ*, *Ifnα4*, *Cxcl10*, *Tnfα*, and *Il6* during HSV‐1 infection (Figure 1H; Figure S1H, Supporting Information). Moreover, the knockdown of *HOIP* abrogated HSV‐1‐induced production of IFN‐β and TNF‐α, whereas *OTULIN* knockdown enhanced the production of IFN‐β and TNF‐α induced by HSV‐1 (Figure S1E,F, Supporting Information). Additionally, immunoblot analysis of HSV‐1‐induced phosphorylation of IRF3 and p65 showed that HOIP promoted the activation of IRF3 and p65, while OTULIN exerted opposite effects during HSV‐1 infection (Figure 1I,J). Consistently, higher viral titers were detected in *HOIP* knockdown cells compared to wild‐type cells, while *OTULIN* knockdown resulted in decreased HSV‐1 titers (Figure 1K,L). In addition, we also detected the expression of *Hoip*, *Hoil‐1l*, *Sharpin*, and *Otulin* in immortalized bone marrow‐derived macrophage (iBMDM) cells and found that the treatment of ISD, but not poly (I:C), induced the elevated expression of *Hoip*, *Hoil‐1l* and *Otulin* (Figure S1I, Supporting Information). Furthermore, we constructed the *Hoip* knockout (*Hoip* ^−/−^) and *Otulin* knockout (*Otulin* ^−/−^) iBMDM cell lines (Figure S1J, Supporting Information). Enzyme‐linked immunosorbent assay (ELISA) and plaque assays showed that HOIP knockout reduced HSV‐1‐induced production of IFNβ and TNFα with increasing virus titers, while OTULIN knockout augmented the production of IFNβ and TNFα induced by HSV‐1 and inhibited viral replication (Figure S1K–N, Supporting Information). Taken together, these results indicate that HOIP and OTULIN bidirectionally regulate immune responses against DNA viruses.

### 2.2 STING Is a Physiological Substrate of Linear Ubiquitination During DNA Virus Infection

**Figure 2:**
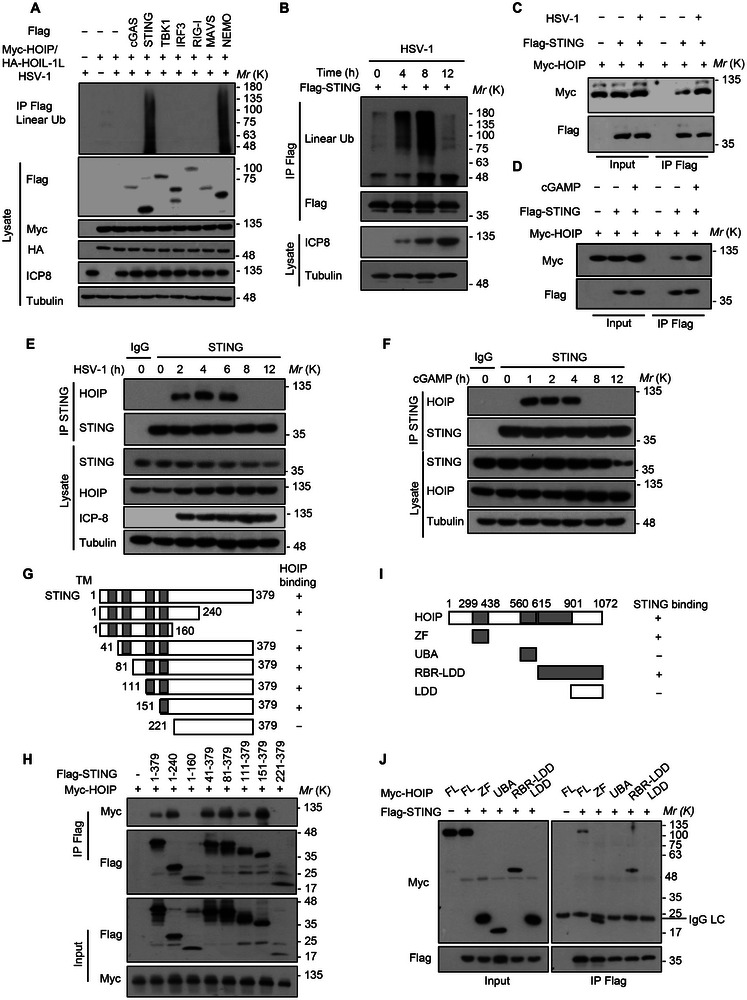
STING is a physiological substrate of linear ubiquitination during DNA virus infection. A) Immunoblot analysis of the linear polyubiquitination of cGAS, STING, TBK1, IRF3, RIG‐I, MAVS, or NEMO in HEK293T cells transfected with indicated plasmids using Lipo2000, followed by infected with HSV‐1 for 6 h. B) Immunoblot analysis of the linear polyubiquitination of STING in HEK293T cells infected with HSV‐1 for the indicated times. C,D) Immunoprecipitation analysis of the interaction between STING and HOIP in HEK293T cells cotransfected with Flag‐STING and Myc‐HOIP using Lipo2000, followed by infected with HSV‐1 for 6 h (C) or 2 µg/mL cGAMP for 4 h (D). E and F) Immunoprecipitation analysis of the interaction between STING and HOIP in THP‐1 cells infected with HSV‐1 for the indicated time points (E), or stimulated with 2 µg/mL cGAMP for the indicated time points (F). G and H) A schematic diagram of STING truncations (G) and immunoprecipitation analysis of the interaction between STING domains and HOIP in HEK293T cells cotransfected with Flag‐STING truncations and Myc‐HOIP using Lipo2000 (H). I and J) A schematic diagram of HOIP truncations (I) and immunoprecipitation analysis of the interaction between HOIP truncations and STING in HEK293T cells cotransfected with Myc‐HOIP truncations and Flag‐STING using Lipo2000 (J). ZF, zinc finger; UBA, ubiquitin‐associated domain; RBR, RING‐in‐between‐RING; LDD, linear ubiquitin chain‐determining domain. Data are representative of three independent experiments.

To assess whether the enzymatic activity of HOIP is involved in the antiviral immune responses against DNA viruses, THP‐1 cells were pretreated with compound 11a, a specific inhibitor of HOIP enzyme activity.^[^ ^35^ ^]^ The results showed that the treatment of compound 11a inhibited HSV‐1‐induced expression of *Ifnβ* and *Ifnα4* (Figure S2A, Supporting Information). ELISA and immunoblot analysis also showed that 11a treatment reduced the production of IFNβ as well as phosphorylation of IRF3 and TBK1 induced by HSV‐1 infection (Figure S2B–D, Supporting Information). Meanwhile, the titer of HSV‐1 virus was significantly increased in 11a‐treated THP‐1 cells compared with mock‐treated group (Figure S2E, Supporting Information). Furthermore, by reintroducing wild‐type HOIP or catalytically inactive HOIP C885S into *Hoip* ^−/‐^ iBMDM cells, we demonstrated that HOIP catalytic activity was required for HSV‐1‐induced IFNβ production (Figure S2F, Supporting Information). These results indicate that LUBAC promotes anti‐DNA virus immune responses in a linear ubiquitination‐dependent manner. We next sought to identify candidate substrates for linear ubiquitination during DNA virus infection. Linear ubiquitination assays of key antiviral signaling proteins including cGAS, STING, TBK1, IRF3, retinoic acid‐inducible gene‐I (RIG‐I), mitochondrial antiviral signaling protein (MAVS) were performed upon HSV‐1 infection, the results showed that STING was specifically linear ubiquitinated by LUBAC (**Figure** **2**A; Figure S2G, Supporting Information). Moreover, the catalytically inactive HOIP C885S mutant failed to promote the linear ubiquitination of STING (Figure S2H, Supporting Information). It is well established that STING could be activated by 2’3’‐cyclic GMP‐AMP (cGAMP), which was synthesized by cGAS in response to double‐strand DNA in the cytoplasm.^[^ ^4^ ^]^ Notably, HSV‐1 infection or cGAMP stimulation induced increased linear ubiquitination of STING, which could be inhibited by compound 11a (Figure 2B; Figure S2I,J, Supporting Information). Co‐immunoprecipitation (Co‐IP) assays further confirmed that the interaction of STING and HOIP was increased following HSV‐1 infection or cGAMP stimulation (Figure 2C–F), suggesting that the activation of STING by upstream signals triggers its linear ubiquitination. STING is an ER resident protein containing three structural domains: four N‐terminal transmembrane helices (amino acids 1–154), a central globular domain (amino acids 155–341), and a cytoplasmic C‐terminal tail (amino acids 342–379).^[^ ^36^ ^]^ To determine which domain of STING is necessary for the interaction with HOIP, we constructed several STING deletion mutants and assessed them in Co‐IP experiments, and found that STING interacted with HOIP through its 161–221 amino acid region (Figure 2G,H). HOIP is composed of a Npl4 zinc finger (ZF) domain, a ubiquitin‐associated (UBA) domain, a RING‐in‐between‐RING (RBR), and a linear ubiquitin chain‐determining domain (LDD) (Figure 2I). Co‐IP experiments demonstrated that the ZF and RBR domains of HOIP were involved in the interaction with STING (Figure 2J). Although previous studies have reported that linear ubiquitination of IRF3 is induced at late stage of RNA virus infection, in our study, the linear ubiquitination of IRF3 was hardly observed during DNA virus infection while linear ubiquitination of STING was obviously upregulated at the early stage of DNA virus infection (Figure S2K,L, Supporting Information). Therefore, STING is a physiological substrate of linear ubiquitination involved in DNA virus infection.

### 2.3 Linear Ubiquitination of STING at Lysine 338 Is Essential for Antiviral Immune Responses

**Figure 3:**
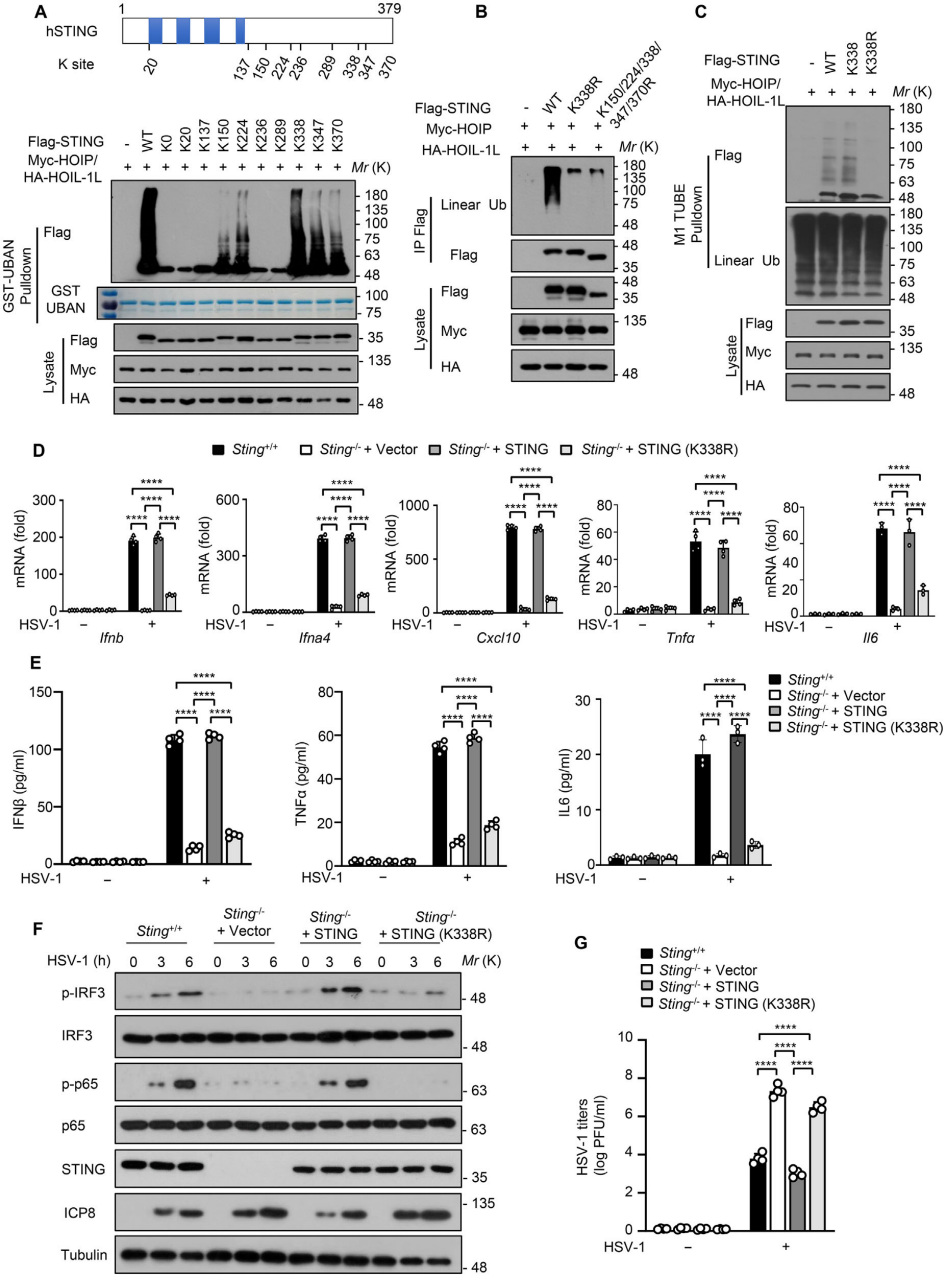
Linear ubiquitination of STING at lysine 338 is essential for antiviral immune responses. A) Immunoblot analysis of the linear polyubiquitination of STING in HEK293T cells transfected with Flag‐STING or its K‐only mutants using Lipo2000. GST‐tagged UBAN used to isolate linear Ub chains were subjected to SDS‐polyacrylamide gel for coomassie brilliant blue staining. B) Immunoblot analysis of the linear polyubiquitination of STING in HEK293T cells cotransfected with Flag‐STING, Flag‐STING (K338R) or Flag‐STING (K150/224/338/347/370R) mutant using Lipo2000. C) Immunoblot analysis of the linear polyubiquitination of STING in HEK293T cells cotransfected with Flag‐STING, Flag‐STING (K338) or Flag‐STING (K338R) using Lipo2000. D) qRT‐PCR analysis of *Ifnb*, *Ifna4*, *Cxcl10*, *Tnfα*, *Il6* mRNA in *Sting* ^−/‐^ iBMDM cells stably expressing WT STING or STING (K338R) infected with HSV‐1 for 6 h. E) ELISA analysis of IFNβ, TNFα and IL6 in *Sting* ^−/‐^ iBMDM cells stably expressing WT STING or STING (K338R) mutant infected with HSV‐1 for 6 h. F) Immunoblot analysis of the p‐IRF3, IRF3, p‐p65, p65, ICP8, STING or Tubulin proteins in *Sting* ^−/−^ iBMDM cells stably expressing WT STING or STING (K338R) infected with HSV‐1 for indicated time points. G) Plaque assay of HSV‐1 titers in *Sting* ^−/−^ iBMDM cells stably expressing WT STING or STING (K338R) infected with HSV‐1 for 6 h. Data are presented as the mean ± SD. Statistical significance was determined by two‐way ANOVA with Tukey's multiple comparisons test (D, E, and G). ****P < 0.0001. Data are representative of three independent experiments.

STING contains nine lysine (K) residues according to the protein sequence analysis (**Figure** **3**A). Multiple lysine residues in the STING protein are known to serve as the sites for ubiquitination, including K20, K150, K224 and K236.^[^ ^37^ ^]^ In order to identify the linear ubiquitination sites of STING, K0 mutant (all nine K residues in STING were mutated to arginine) and K‐only mutants (all K residues in STING were mutated to arginine except for the indicated K sites) were constructed and we found that STING K150, K224, K338, K347 and K370 were potential linear ubiquitination sites modified by LUBAC (Figure 3A). By constructing the STING KR mutants of these sites and performing linear ubiquitination assay, we demonstrated that K150/224/338/347/370R mutant abrogated the linear ubiquitination of STING to a similar extent as the K338R mutant (Figure 3B). Consistently, K338 could be efficiently linear‐ubiquitinated by LUBAC, while the K338R mutant completely abolished the linear ubiquitination of STING (Figure 3C). Moreover, mass spectrometry also identified that K338 was the linear ubiquitinated site of STING during HSV‐1 infection and HOIP knockdown decreased the ubiquitin level on the K338 sites of STING (Figure S3A,B, Supporting Information). Although K370 residue of STING was identified as a potential linear ubiquitination site by mass spectrometry (Figure S3C, Supporting Information), K370R mutant did not affect the linear ubiquitination of STING (Figure S3D, Supporting Information). This result further suggests that K338 was the major linear ubiquitination site of STING. Previous studies have reported that STING could be sumoylated, ISGylated, and acetylated at K338 site,^[^ ^38^, ^39^, ^40^ ^]^ we searched the mass spectrometry data and no STING K338 acetylation was detected. We also detected the sumoylation and ISGylation of STING and found that STING was not modified by sumoylated and ISGylated in our experimental conditions (Figure S3D, Supporting Information). Furthermore, we demonstrated that K338R mutant abolished STING phosphorylation and LUBAC‐mediated linear ubiquitination of STING during HSV‐1 infection and upon cGAMP stimulation (Figure S3E–G, Supporting Information). Additionally, we detected the effects of K338R mutant in antiviral immune response against DNA viruses. We found that the reintroduction of STING (K338R) in *Sting* ^−/−^ iBMDM cells failed to restore HSV‐1‐induced expression of *Ifnb*, *Ifna4*, *Cxcl10*, *Tnfα* and *Il6* (Figure 3D). ELISA assay further showed that the STING (K338R) mutant failed to promote the production of IFNβ, TNFα and IL6 (Figure 3E). Immunoblot analysis also confirmed attenuated phosphorylation of IRF3 and p65 in *Sting* ^−/−^ iBMDM cells expressing STING (K338R) (Figure 3F). Consistent with that, the restoration of STING (K338R) in *Sting* ^−/−^ iBMDM cells resulted in elevated viral replication after infection with HSV‐1 (Figure 3G). Collectively, these data suggest that the linear ubiquitination of STING at lysine 338 is essential for antiviral immune responses against DNA viruses.

### 2.4 Linear Ubiquitin Chain Conjugated on STING Recognizes Sec24b ZF Domain to Promote the ER‐to‐Golgi Trafficking of STING

**Figure 4:**
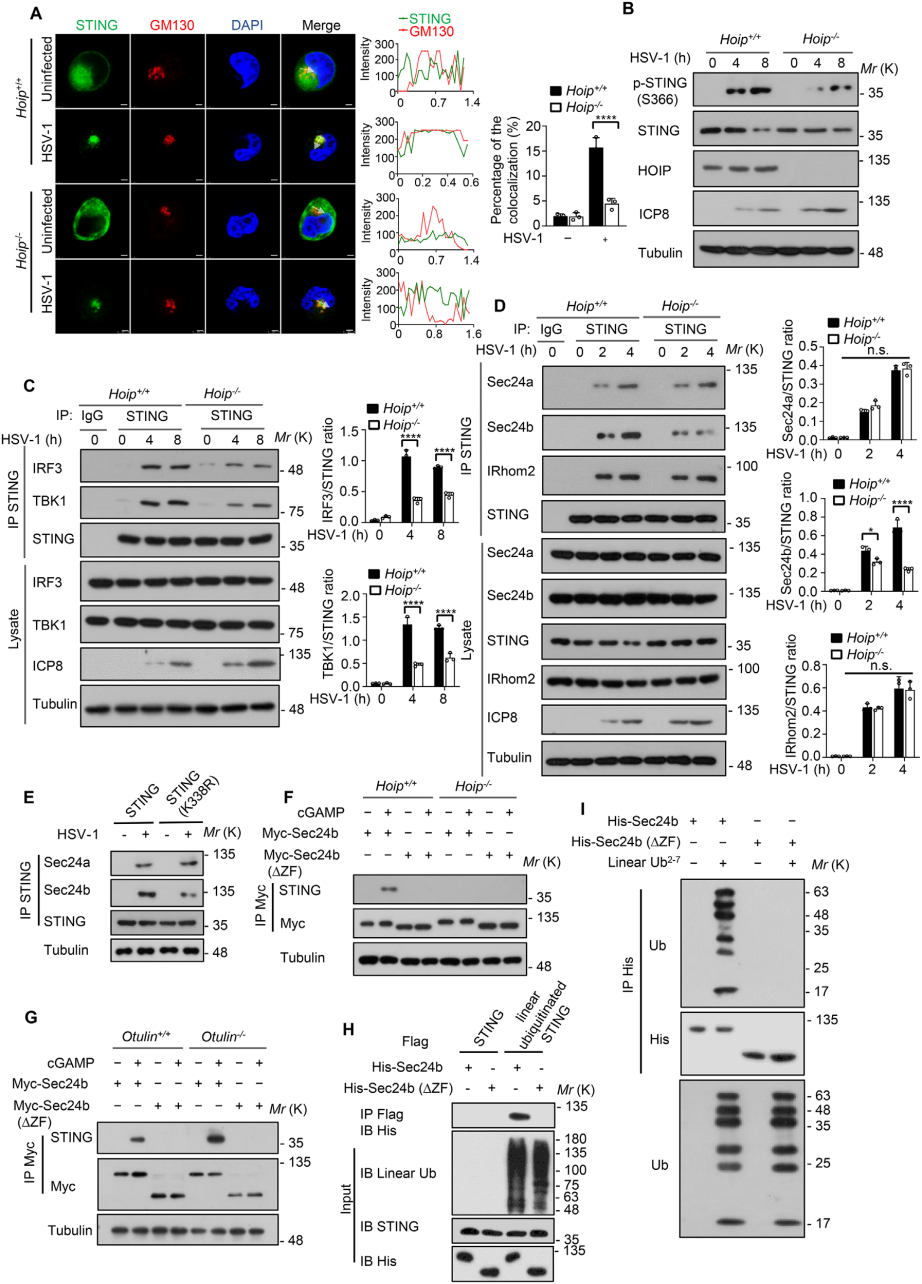
Linear ubiquitination of STING promotes its trafficking from the ER to the Golgi apparatus depending on Sec24b. A) Immunofluorescence analysis of the colocalization of endogenous STING and GM130 in *Hoip* ^+/+^ and *Hoip* ^−/‐^ cells infected with HSV‐1 for 4 h. Scale bars, 10 µm. Relative fluorescence intensities of STING and GM130 were measured using Image J along the arrows in the middle. The quantitated colocalization was determined by fluorescence intensities. The percentage of colocalization of STING with Golgi marker GM130 is shown on the right. About 100 cells were counted and analyzed for each biological replicate. B) Immunoblot analysis of the phosphorylated STING, total STING, HOIP, ICP8 and Tubulin in *Hoip* ^+/+^ and *Hoip* ^−/‐^ iBMDM cells infected with HSV‐1 for the indicated time points. C) Immunoblot analysis of the interaction between endogenous STING and TBK1 or IRF3 in *Hoip* ^+/+^ and *Hoip* ^−/‐^ iBMDM cells infected with HSV‐1 for the indicated time points. The densitometry quantitative analysis of IRF3 or TBK1 relative to STING are shown on the right. D) Immunoblot analysis of the interaction between endogenous STING and Sec24a, Sec24b, or IRhom2 in *Hoip* ^+/+^ and *Hoip* ^−/‐^ iBMDM cells infected with HSV‐1 for the indicated time points. The densitometry quantitative analysis of Sec24a, Sec24b or IRhom2 relative to STING are shown on the right. E) Immunoblot analysis of the interaction between STING, and Sec24a or Sec24b in HEK293T cells transfected with Flag‐STING or Flag‐STING (K338R) using Lipo2000. F) Immunoblot analysis of the interactions between transfected Myc‐Sec24b or Myc‐Sec24b (∆ZF) and endogenous STING in *Hoip* ^+/+^ and *Hoip* ^−/‐^ iBMDM cells treated with 2 µg/mL cGAMP for 2 h. G) Immunoblot analysis of the interactions between transfected Myc‐Sec24b or Myc‐Sec24b (∆ZF) and endogenous STING in *Otulin* ^+/+^ and *Otulin* ^−/‐^ iBMDM cells treated with 2 µg/mL cGAMP for 2 h. H) Immunoblot analysis of the interactions of linear ubiquitinated STING between Sec24b and Sec24b (∆ZF). Linear ubiquitinated STING was isolated from HEK 293T transfected with Flag‐tagged STING, Myc‐tagged HOIP, HA‐tagged HOIL‐1L, and subsequently incubated with BL21‐purified His‐Sec24b or His‐Sec24b (∆ZF) proteins overnight at 4 °C, followed by immunoblot analysis of the interactions. STING purified in HOIP knock‐down cells was a negative control without linear ubiquitination. I) His‐Sec24b, His‐Sec24b (∆ZF) and linear ubiquitin chain was incubated at 37 °C for 1 h, followed by immunoblot analysis of the interactions. Data are presented as the mean ± SD. Statistical significance was determined by two‐way ANOVA with Sidak's multiple comparisons test (A, C, D). *P < 0.05, ****P < 0.0001. Data are representative of three independent experiments.

The activation of STING signaling pathway requires cGAMP binding, STING polymerization, ER‐to‐Golgi trafficking of STING, STING phosphorylation, and the recruitment of TBK1 and IRF3 by STING.^[^ ^41^ ^]^ To pinpoint which step of STING activation was regulated by linear ubiquitination, we stimulated WT and *Hoip* knockout cells with biotin‐tagged cGAMP and subjected them to immunoprecipitation with streptavidin agarose, the results showed that HOIP knockout did not affect the binding of STING to cGAMP (Figure S4A, Supporting Information). However, HOIP knockout interfered with the homodimerization and oligomerization of STING, as revealed by native polyacrylamide gel electrophoresis (PAGE) (Figure S4B, Supporting Information). Furthermore, we performed immunofluorescence assay with markers for endoplasmic reticulum (Calnexin) and Golgi apparatus (GM130). We found that HOIP colocalized with STING in endoplasmic reticulum, and HOIP knockout promoted STING retention in the endoplasmic reticulum and attenuated the localization of STING in Golgi apparatus following HSV‐1 infection (**Figure** **4**A; Figure S4C–F, Supporting Information). Accordingly, the phosphorylation of STING by TBK1 was also reduced in *Hoip* knockout cells infected with HSV‐1 virus (Figure 4B), which was accompanied by the decreased formation of the STING‐TBK1‐IRF3 complex (Figure 4C). Consistently, STING (K338R) mutant also attenuated the colocalization of STING with GM130 (Figure S4G,H, Supporting Information). These data suggest that the linear ubiquitination of STING by LUBAC promotes its trafficking from the ER to the Golgi apparatus.

A variety of proteins have been implicated in mediating the vesicular transport of STING to Golgi apparatus, including COPII, YIPF5, and iRhom2.^[^ ^6^, ^9^, ^11^, ^13^ ^]^ To further elucidate the mechanisms of linear ubiquitination‐mediated STING trafficking to the Golgi apparatus, Co‐IP assay with anti‐STING antibody was performed. The results showed that HOIP knockout attenuated the interaction of STING with COPII subunit Sec24b, but not with COPII subunit Sec24a and iRhom2 (Figure 4D). Previous studies have reported that Sec24c plays a critical role in STING pathway activation,^[^ ^6^ ^]^ we also demonstrated that HOIP deficiency didn't hinder STING's interaction with Sec24c (Figure S5A, Supporting Information). Moreover, the mutation of lysine 338 to arginine and the treatment of HOIP inhibitor 11a also weakened the interaction of STING with Sec24b (Figure 4E; Figure S5B, Supporting Information), indicating that linear ubiquitination of STING by LUBAC facilitates its binding to Sec24b.

By performing sequence analysis using the universal protein resource (Uniprot) database, we found that Sec24b contained a zinc finger‐like domain (amino acids 605–629) (Figure S5C, Supporting Information). Previous studies have reported that zinc finger‐domain containing proteins have the ability to bind ubiquitin chains,^[^ ^42^, ^43^ ^]^ we thus postulated that the zinc finger‐like domain of Sec24b might be involved in the linear ubiquitination‐mediated STING trafficking from the ER to the Golgi apparatus. To test this hypothesis, we performed immunoprecipitation assays to investigate interactions between STING and Sec24b or Sec24b (∆ZF). The results showed that HOIP knockout and 11a treatment also abolished the interactions between STING and Sec24b while OTULIN knockout increased this interaction (Figure 4F,G; Figure S5D, Supporting Information), indicating that linear ubiquitination of STING facilitates its binding to the zinc finger‐like domain of Sec24b. Furthermore, purified Sec24b or Sec24b (∆ZF) proteins were used to test the interaction with linear ubiquitin chains, and the results showed that linear ubiquitin chains and linear ubiquitinated STING both interacted with Sec24b depending on its zinc finger‐like domain (Figure 4H,I). Additionally, the immunofluorescence and immunoblot analysis showed that the deletion of Sec24b ZF domain didn't affect its ability to associate with the COPII complex (Figure S5E,F, Supporting Information). Furthermore, we performed immunofluorescence assay to detect STING trafficking in Sec24b‐deficient iBMDM cells and demonstrated that Sec24b knockout or deletion of Sec24b ZF domain attenuated the colocalization of STING with GM130 following cGAMP stimulation (Figure S5G,H, Supporting Information). Moreover, immunoblot analysis showed that Sec24b knockout or deletion of Sec24b ZF domain didn't affect the linear ubiquitination of STING and the interaction between HOIP and STING, but weakened the binding of STING to OTULIN following cGAMP stimulation (Figure S5I, Supporting Information). These data suggest that Sec24b binds linear ubiquitin chains via its zinc finger‐like domain, thereby facilitating its interaction with linear ubiquitinated STING. Together, these results demonstrate that linear ubiquitination of STING by LUBAC promotes its trafficking from the ER to the Golgi apparatus through binding to the vesicle trafficking protein Sec24b.

### 2.5 OTULIN Is Recruited to Phosphorylated STING and Removes Its Linear Ubiquitin Chains

**Figure 5:**
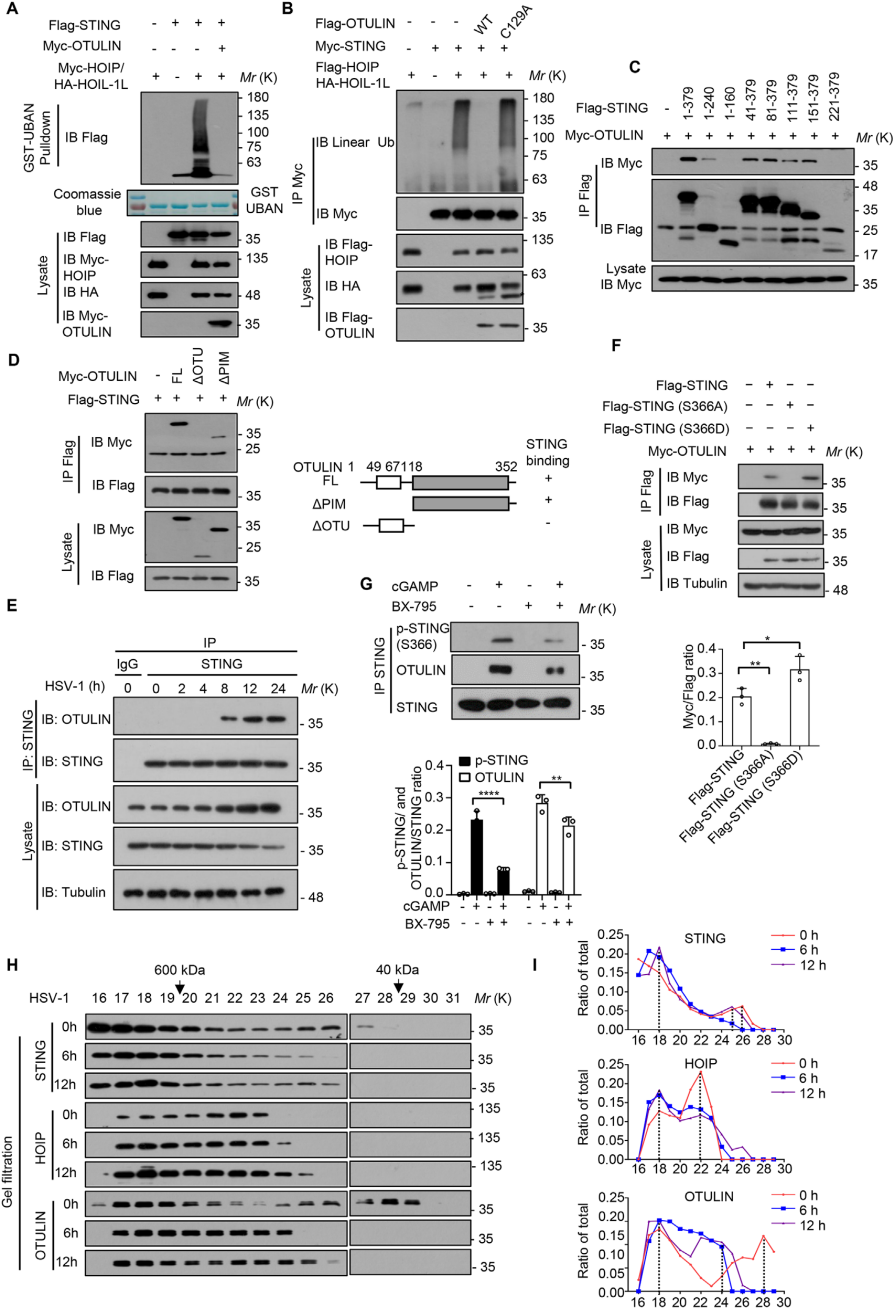
OTULIN is recruited by phosphorylated STING to remove the linear ubiquitin chains from STING. A) Immunoblot analysis of STING linear ubiquitination with or without OTULIN overexpression. GST‐tagged UBAN proteins used to isolate linear Ub chains were subjected to SDS‐polyacrylamide gel for coomassie brilliant blue staining. B) Immunoblot analysis of the deubiquitinating assay of STING by OTULIN or OTULIN (C129A). C) Immunoprecipitation analysis of the interaction between STING domains and OTULIN in HEK293T cells cotransfected with Flag‐STING truncations and Myc‐OTULIN using Lipo2000. D) Immunoprecipitation analysis of the interaction between OTULIN mutants and STING in HEK293T cells cotransfected with Myc‐OTULIN mutants and Flag‐STING using Lipo2000. A schematic diagram of OTULIN mutants on the right. PIM, PNGase/UBA or UBX‐containing proteins (PUB)‐interacting motif; OTU, ovarian tumor. E) Immunoprecipitation analysis of the interaction between endogenous STING and OTULIN in THP‐1 cells infected with HSV‐1 for the indicated time points. F) Immunoblot analysis of the interaction of OTULIN with WT STING, STING (S366A), and STING (S366D). The densitometry quantitative analysis of OTULIN relative to STING or its mutants is shown at the bottom. G) Immunoblot analysis of the phosphorylation of STING and interaction of STING with OTULIN in THP‐1 cells stimulated with 2 µg/mL cGAMP for 8 h in the absence or presence of TBK1 inhibitor 1 µM BX‐795 for 1 h. The densitometry quantitative analysis of p‐STING or OTULIN relative to STING is shown at the bottom. H) Gel filtration profiles of HOIP, OTULIN or STING in HEK293T cells infected by HSV‐1 for the indicated times. Fractions from lysates separated by Superdex 200 HR were subjected to SDS‐PAGE, followed by immunoblotting with indicated antibodies and analysis in (I). I) The ratio distribution of HOIP, OTULIN, or STING (relative to total protein per group) in HEK293T cells infected with HSV‐1 for the indicated times. Data are representative of three independent experiments. Data are presented as the mean ± SD. Statistical significance was determined by Ordinary one‐way comparisons test (F) and two‐way ANOVA with Sidak's multiple comparisons test (G). *P < 0.05, **P < 0.01, ****P < 0.0001. Data are representative of three independent experiments.

Deubiquitinase OTULIN has been known to negatively regulate M1‐linked polyubiquitin signaling through removing the ubiquitin chains conjugated by LUBAC, and OTULIN deficiency could cause OTULIN‐related autoinflammatory syndrome (ORAS) in humans,^[^ ^44^ ^]^ indicating a critical role of OTULIN in maintaining immune homeostasis. Our data described above have demonstrated that OTULIN negatively regulates DNA virus‐induced innate immune responses. By reintroducing wild‐type OTULIN or enzymatically inactive mutant C129A into *Otulin* ^−/−^ iBMDM cells, we found that OTULIN catalytic activity was required for HSV‐1‐induced IFN‐β production (Figure S6A, Supporting Information), indicating that OTULIN negatively regulates anti‐DNA virus immune responses depending on its deubiquitinase activity. Furthermore, we performed in vitro deubiquitination assay with glutathione S‐transferase (GST)‐tagged ubiquitin‐binding in ABIN and NEMO (UBAN) containing the UBAN region of four human NEMO, a tool for linear ubiquitin chains enrichment with high specificity and affinity,^[^ ^45^ ^]^ and confirmed that OTULIN deubiquitinated the LUBAC‐mediated linear ubiquitination of STING (**Figure** **5**A). We also found that WT OTULIN, but not its enzymatically inactive mutant C129A removed the linear ubiquitin chains of STING generated by LUBAC (Figure 5B). By using purified OTULIN or OTULIN (C129A) proteins, we further demonstrated that OTULIN specifically removed the linear polyubiquitin chains from STING in vitro (Figure S6B, Supporting Information). Moreover, the results from Co‐IP and pull‐down assays showed that OTULIN could interact directly with STING independently of its enzymatic activity (Figure S6C,D, Supporting Information). To map the interaction domains of STING involved in the interaction with OTULIN, we performed Co‐IP experiments and demonstrated that the 161–221 fragment of STING was required for binding to OTULIN (Figure 5C). OTULIN harbors a N‐terminal PNGase/UBA or UBX‐containing proteins (PUB)‐interacting motif (PIM) domain and a C‐terminal OTU domain. The PIM domain is responsible for the interaction with HOIP, while the OTU domain mediates the deubiquitinase activity of OTULIN.^[^ ^46^ ^]^ Immunoblot analysis revealed that the OTU domain of OTULIN was required for the interaction with STING (Figure 5D). To confirm that OTULIN targets STING during HSV‐1 infection, Co‐IP assay with anti‐STING antibody was performed and immunoblot analysis demonstrated that OTULIN could bind to STING after HSV‐1 infection or cGAMP stimulation for 8 h (Figure 5E; Figure S6E, Supporting Information), suggesting a critical role of OTULIN in the late stage of HSV‐1 infection.

Next, we sought to explore the mechanism of how OTULIN is recruited to STING in the late stage of HSV‐1 infection. Previous study has reported OTULIN could bind to HOIP through its PIM domain,^[^ ^47^ ^]^ we thus postulated that OTULIN could be recruited by HOIP to interact with STING and remove the linear ubiquitination of STING in the late stage of HSV‐1 infection. A co‐immunoprecipitation experiment has been performed to ascertain the interactions between STING and OTULIN or its truncation mutants in wild‐type or HOIP‐deficient HEK293T cells. Our results showed that HOIP deficiency did not affect the interactions between STING and OTULIN or its truncation mutants (Figure S6F, Supporting Information), indicating that OTULIN is not recruited to STING via HOIP. After transport from the ER to the Golgi apparatus, STING is phosphorylated by TBK1 to induce the formation of STING signalosome, which serves as a platform to trigger antiviral immune responses.^[^ ^48^ ^]^ We thus speculated that TBK1‐mediated STING phosphorylation might promote the recruitment of OTULIN in the later stage of DNA virus infection to terminate STING signaling in a feedback manner. To examine this hypothesis, we constructed STING phosphomimetic and non‐phosphorylatable mutants by substituting a serine at position 366 with an aspartate (S366D) or an alanine (S366A), respectively. We found that STING (S366D) displayed an elevated interaction with OTULIN, whereas STING (S366A) lost its ability to bind to OTULIN (Figure 5F). Moreover, activated STING phosphorylation at Ser366 was detected along with enhanced STING‐OTULIN interaction at the same time points following cGAMP treatment, which effects were attenuated by BX‐795, a specific TBK1 inhibitor (Figure 5G), suggesting that the phosphorylation of STING at S366 is indispensable for STING‐OTULIN interaction. To further examine whether STING recruits OTULIN after its vesicle trafficking, we pretreated iBMDM cells with brefeldin A (BFA), an Arf1 GTPase inhibitor that blocks STING ER‐to‐Golgi transport. Our results showed that BFA treatment disrupted the specific interaction of STING with OTULIN, but not with HOIP (Figure S6G–J, Supporting Information), indicating that STING interacts with OTULIN after its vesicle trafficking, whereas STING binds to HOIP prior to its ER‐to‐Golgi translocation.

Furthermore, we performed fractionation experiments to verify the specific interactions among HOIP, OTULIN, and STING during HSV‐1 infection. Whole cell lysates were fractionated by fast protein liquid chromatography (FPLC) followed by immunoblotting of the fractions for STING, HOIP, and OTULIN. Upon HSV‐1 stimulation at 6 h, ≈5% STING shifted from lower molecular weight fractions (around fraction 26) to higher molecular weight fractions (around fraction 18) due to the fact that ∼10% HOIP in lower molecular weight fractions (around fraction 22) resided in fraction 18 with STING. Upon HSV‐1 stimulation at 12 h, despite that HOIP distribution in fraction 18 remained almost unchanged, ≈5% STING resided in lower molecular weight fractions (around fraction 25). This was probably due to the fact that ≈8% OTULIN bound to STING and resided in lower molecular weight fractions (around fraction 25) (Figure 5H,I). These data suggest that HOIP binds to STING around 6 h after HSV‐1 infection and then OTULIN interacts with STING to dissociate STING from higher molecular weight complex at 12 h of HSV‐1 infection. Moreover, we detected the interactions between STING, HOIP, OTULIN, and Sec24b at the different time points following cGAMP stimulation (Figure S6K, Supporting Information). Our results showed that STING interacted with HOIP and Sec24b at the early stage of HSV‐1 infection and then bound to OTULIN at the late stage of the infection. Additionally, we also detected the interactions between STING or STING (S366D) mutant and HOIP or OTULIN. Our results showed that STING (S366D) mutant enhanced the interaction between STING and OTULIN, while weakening the interaction between STING and HOIP (Figure S6L, Supporting Information), indicating that the phosphorylation of STING (S366) site functions as a switch to regulate the interactions between STING and HOIP or OTULIN at the different stages of viral infection. We also detected the interactions between WT STING (or STING K338R mutant) and HOIP or OTULIN during HSV‐1 infection, and the results showed that STING K338R mutation attenuated the binding of STING to OTULIN, but not to HOIP (Figure S7A, Supporting Information), further confirmed that the linear ubiquitination dependent phosphorylation of STING is crucial for STING‐OTULIN interaction.

Next, we further explore the regulatory function of STING by OTULIN. Immunofluorescence assay and immunoblot analysis demonstrated that OTULIN colocalized with STING in Golgi apparatus upon virus infection, and the overexpression of wild‐type OTULIN decreased the localization of STING in Golgi apparatus following cGAMP stimulation, while the knockout of OTULIN enhanced the localization of STING in Golgi apparatus (Figure S7B–G, Supporting Information). Consistently, the oligomerization of STING was increased in *Otulin* ^−/−^ iBMDM cells (Figure S8A, Supporting Information), suggesting that OTULIN promotes the translocation of STING in Golgi apparatus. Previous studies have shown that STING could translocate from the Golgi apparatus to the endolysome, and the subsequent termination of STING signaling is regulated by the Golgi exit regulator GCC2,^[^ ^49^ ^]^ clathrin‐coated vesicles (CCVs)^[^ ^50^, ^51^ ^]^ and ESCRT complexes.^[^ ^52^, ^53^, ^54^ ^]^ We thus examined whether OTULIN regulates the degradation of STING. Our results showed that OTULIN promoted STING degradation during HSV‐1 infection (Figure S8B, Supporting Information). Moreover, OTULIN‐mediated degradation of STING can be inhibited by V‐ATPase inhibitor bafilomycin A1 (Figure S8C, Supporting Information). Furthermore, we detected the interaction of STING with the Golgi exit regulator GCC2, AP1S1 in clathrin‐coated vesicles (CCVs) as well as HGS and VPS37A in ESCRT complexes. Our results showed that OTULIN deficiency could attenuate the interaction of STING and AP1S1 (Figure S8D, Supporting Information) and knockout of AP1S1 resulted in a marked increase in the levels of STING (Figure S8E, Supporting Information). Consistently, the mutant STING K338R (which has decreased linear ubiquitination and restricted location in Golgi apparatus) showed weakened interaction with AP1S1 when compared with wild‐type STING (Figure S8F, Supporting Information). These results indicated that OTULIN facilitated lysosome‐mediated STING degradation through clathrin‐coated vesicles. Taken together, OTULIN could be recruited by phosphorylated STING to remove the linear ubiquitin chains on STING at the late stage of virus infection, thus promoting lysosome‐mediated STING degradation to control antiviral immune responses against DNA virus.

### 2.6 HOIP Promotes, While OTULIN Inhibits, Antiviral Immune Responses Against DNA Virus In Vivo

To evaluate the physiological functions of HOIP and OTULIN in anti‐DNA virus innate immune responses, we constructed *Hoip* or *Otulin* myeloid cell‐specific knockout mice by crossing *Hoip^loxP/loxP^* or *Otulin^loxP/loxP^* mice with *Lyz‐Cre* mice. The successful conditional knockout of *Hoip* or *Otulin* was confirmed by detection of HOIP or OTULIN protein expression in BMDMs (Figure S9A,B, Supporting Information). As expected, HSV‐1 infection upregulated the expression of *Hoip*, *Hoil‐1l*, and *Otulin* in BMDMs (Figure S9C, Supporting Information).

**Figure 6:**
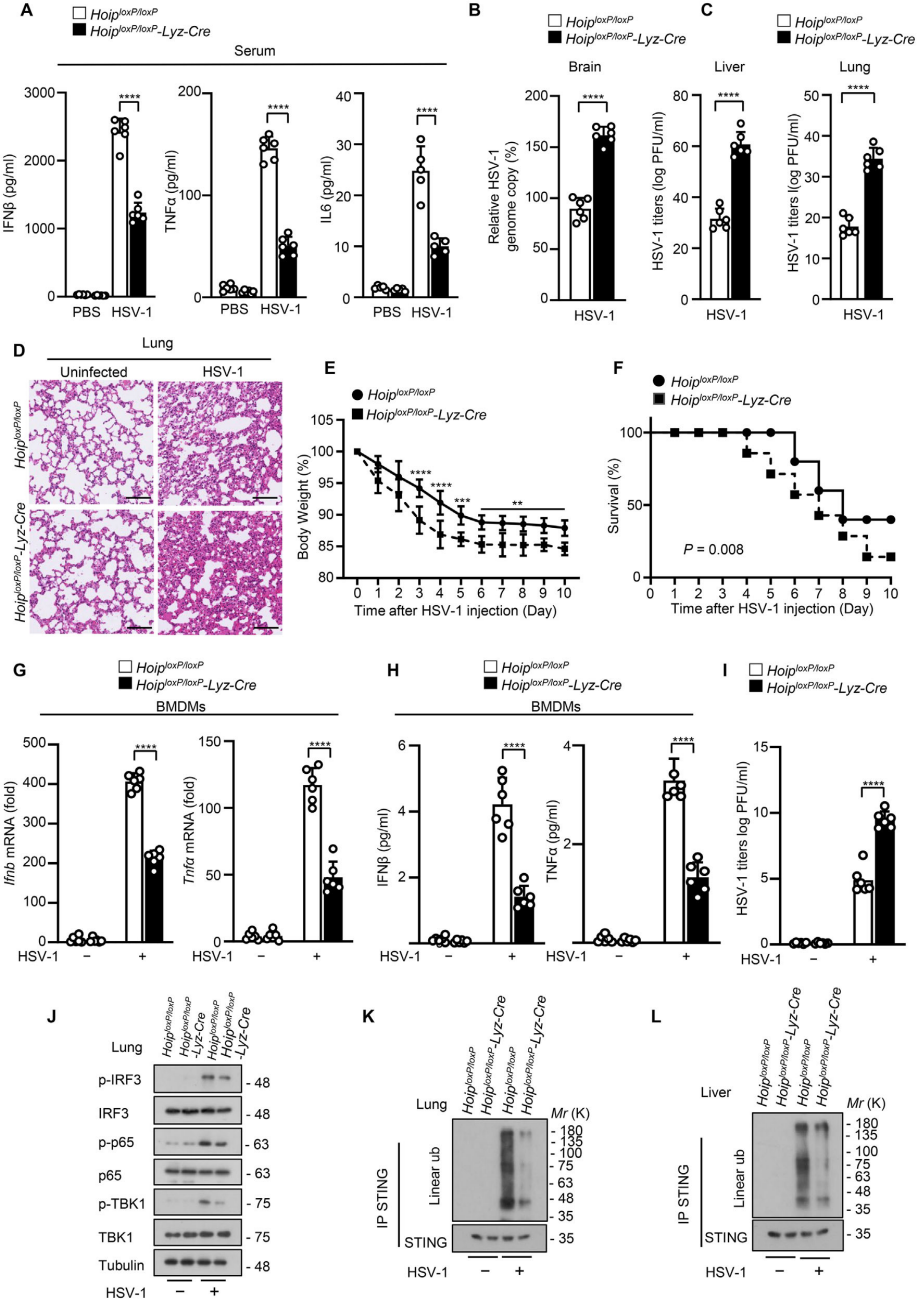
HOIP promotes antiviral innate immune responses against HSV‐1 in vivo. A) ELISA analysis for IFNβ, TNFα, and IL6 secretion in sera from *Hoip^loxP/loxP^* and *Hoip^loxP/loxP^‐Lyz‐Cre* mice intravenously infected with HSV‐1 for 24 h. B) Viral titers in homogenates of brains from *Hoip^loxP/loxP^* and *Hoip^loxP/loxP^*‐*Lyz‐Cre* mice (*n* = 6) after intravenous injection of HSV‐1 (1 × 10^6^ PFU per mouse). C) Plaque assays analysis of HSV‐1 titers in the livers or lungs from *Hoip^loxP/loxP^* and *Hoip^loxP/loxP^‐Lyz‐Cre* mice intravenously infected with HSV‐1 for 3 days. D) Hematoxylin‐eosin staining of lungs of *Hoip^loxP/loxP^* and *Hoip^loxP/loxP^*‐*Lyz‐Cre (n* = 6) after intravenous injection of HSV‐1 (1 × 10^6^ PFU per mouse). Scale bars, 50 µm. (E) Body weight of *Hoip^loxP/loxP^* and *Hoip^loxP/loxP^*‐*Lyz‐Cre* mice (*n* = 6) after intravenous injection of HSV‐1 (1 × 10^6^ PFU per mouse). F) Survival (Kaplan‐Meier curves) of *Hoip^loxP/loxP^* and *Hoip^loxP/loxP^*‐*Lyz‐Cre* mice (*n* = 6) after intravenous injection of HSV‐1 (1 × 10^6^ PFU per mouse). G) qRT‐PCR analysis of *Ifnb* and *Tnfα* mRNA in *Hoip^loxP/loxP^* and *Hoip^loxP/loxP^*‐*Lyz‐Cre* BMDMs infected with HSV‐1 for 8 h. H) ELISA analysis for IFNβ or TNFα secretion in *Hoip^loxP/loxP^* and *Hoip^loxP/loxP^*‐*Lyz‐Cre* BMDMs infected with HSV‐1 for 12 h. I) Plaque assay of HSV‐1 titers in *Hoip^loxP/loxP^* and *Hoip^loxP/loxP^*‐*Lyz‐Cre* BMDMs infected with HSV‐1 for 12 h. (J) Immunoblot analysis of p‐TBK1, p‐IRF3, and p‐p65 in the lungs from *Hoip^loxP/loxP^* and *Hoip^loxP/loxP^‐Lyz‐Cre* mice intravenously infected with HSV‐1 for 3 days. K and L) Immunoblot analysis of linear ubiquitination of STING in the lungs (K) or livers (L) from *Hoip^loxP/loxP^* and *Hoip^loxP/loxP^‐Lyz‐Cre* mice intravenously infected with HSV‐1 for 3 days. Data are presented as the mean ± SD. Statistical significance was determined by two‐way ANOVA with Sidak's multiple comparisons test (A, E, G, H and I), unpaired two‐tailed Student's *t*‐tests (B, C) and Log‐rank (Mantel‐Cox) test (F). **P < 0.01, ****P < 0.0001. Data are representative of three independent experiments.

We first investigate the physiological functions of HOIP in the antiviral innate immune responses. We infected *Hoip^loxP/loxP^*‐*Lyz‐Cre* mice and their counterparts with HSV‐1 virus and found that *Hoip^loxP/loxP^*‐*Lyz‐Cre* mice had lower concentrations of IFNβ, TNFα, and IL6 in serum and higher viral titers in the brains, livers and lungs than their littermate controls (**Figure** **6**A–C). Consistent with that, *Hoip^loxP/loxP^*‐*Lyz‐Cre* mice displayed more severe lung injury, higher body weight loss, and lower survival rate compared with *Hoip^loxP/loxP^* mice (Figure 6D–F), suggesting that HOIP depletion in myeloid cells renders mice more susceptible to HSV‐1 virus infection. Subsequently, BMDMs from *Hoip* conditional knockout mice were isolated and infected with HSV‐1, and our results showed that *Hoip* knockout decreased the HSV‐1‐induced production of IFNβ and TNFα, and increased HSV‐1 viral replication (Figure 6G–I). We also detected STING signaling and its linear ubiquitination in tissues from *Hoip^loxP/loxP^*‐*Lyz‐Cre* mice. The levels of p‐IRF3, p‐TBK1, and p‐p65 were both decreased in the lungs of *Hoip^loxP/loxP^*‐*Lyz‐Cre* mice infected with HSV‐1 virus. Consistently, STING linear ubiquitination in the lung and liver of *Hoip^loxP/loxP^*‐*Lyz‐Cre* mice was much lower than those of *Hoip^loxP/loxP^* mice during DNA virus infection (Figure 6J–L). These results suggest that HOIP promotes antiviral immune responses against DNA virus in vivo.

**Figure 7:**
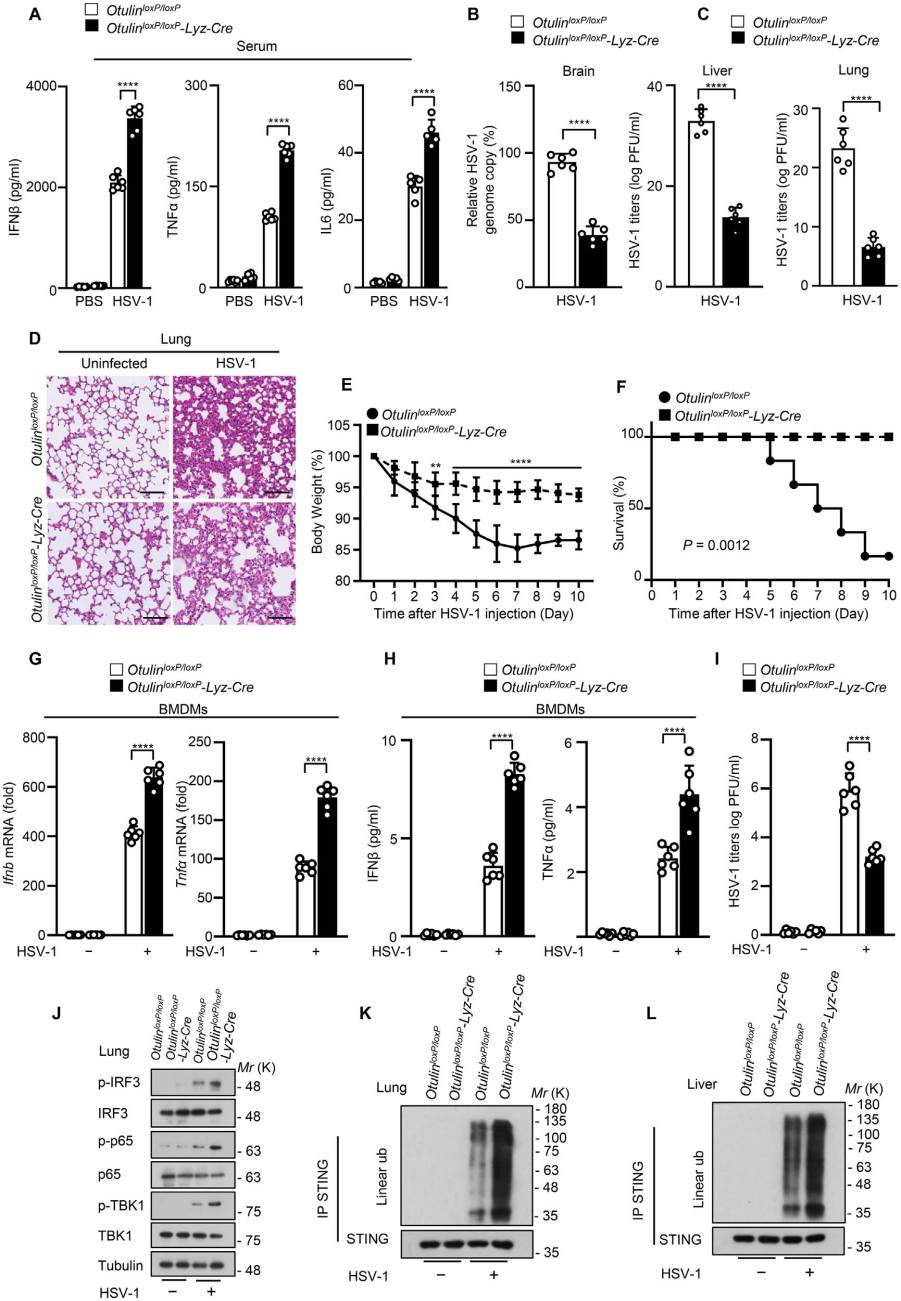
OTULIN inhibits antiviral innate immune responses against HSV‐1 in vivo. A) ELISA analysis for IFNβ or TNFα secretion in sera from *Otulin^loxP/loxP^* and *Otulin ^loxP/loxP^*‐*Lyz‐Cre* mice (*n* = 6) infected with HSV‐1 for 24 h. B) Viral titers in homogenates of brains from *Otulin^loxP/loxP^* and *Otulin ^loxP/loxP^*‐*Lyz‐Cre* mice (*n* = 6) after intravenous injection of HSV‐1 (1 × 10^6^ PFU per mouse). C) Plaque assays analysis of HSV‐1 titers in the liver or lung from *Otulin^loxP/loxP^* and *Otulin ^loxP/loxP^‐Lyz‐Cre* mice intravenously infected with HSV‐1 for 3 days. D) Hematoxylin‐eosin staining of lungs of *Otulin^loxP/loxP^* and *Otulin ^loxP/loxP^*‐*Lyz‐Cre* (*n* = 6) after intravenous injection of HSV‐1 (1 × 10^6^ PFU per mouse). Scale bars, 50 µm. E) Body weight of *Otulin^loxP/loxP^* and *Otulin ^loxP/loxP^*‐*Lyz‐Cre* mice (*n* = 6) after intravenous injection of HSV‐1 (1 × 10^6^ PFU per mouse). F) Survival (Kaplan‐Meier curves) of *Otulin^loxP/loxP^* and *Otulin^loxP/loxP^*‐*Lyz‐Cre* mice (*n* = 6) after intravenous injection of HSV‐1 (1 × 10^6^ PFU per mouse). G) qRT‐PCR analysis of *Ifnb* and *Tnfα* mRNA in *Otulin^loxP/loxP^* and *Otulin^loxP/loxP^*‐*Lyz‐Cre* BMDMs infected with HSV‐1 for 8 h. H) ELISA analysis for IFNβ or TNFα secretion in *Otulin^loxP/loxP^* and *Otulin^loxP/loxP^*‐*Lyz‐Cre* BMDMs infected with HSV‐1 for 12 h. I) Plaque assay of HSV‐1 titers in *Otulin^loxP/loxP^* and *Otulin^loxP/loxP^*‐*Lyz‐Cre* BMDMs infected with HSV‐1 for 12 h. J) Immunoblot analysis of p‐TBK1, p‐IRF3, and p‐p65 in the lungs from *Otulin^loxP/loxP^* and *Otulin^loxP/loxP^‐Lyz‐Cre* mice intravenously infected with HSV‐1 for 3 days. K and L) Immunoblot analysis of linear ubiquitination of STING in the lungs (K) or livers (L) from *Otulin^loxP/loxP^* and *Otulin^loxP/loxP^‐Lyz‐Cre* mice intravenously infected with HSV‐1 for 3 days. Data are presented as the mean ± SD. Statistical significance was determined by two‐way ANOVA with Sidak's multiple comparisons test (A, E, G, H, and I), unpaired two‐tailed Student's *t*‐tests (B, C), and Log‐rank (Mantel‐Cox) test (F). **P < 0.01, ****P < 0.0001. Data are representative of three independent experiments.

Next, the physiological function of OTULIN in anti‐DNA virus innate immune responses was tested. *Otulin^loxP/loxP^*‐*Lyz‐Cre* mice had higher concentrations of IFNβ, TNFα, and IL6 in serum and lower viral titers in the brains, livers, and lungs compared with *Otulin^loxP/loxP^* mice (**Figure** **7**A–C). Meanwhile, *Otulin^loxP/loxP^*‐*Lyz‐Cre* mice exhibited less severe lung injury, lower body weight loss and higher survival rate than their littermate controls (Figure 7D–F), indicating that *Otulin^loxP/loxP^*‐*Lyz‐Cre* mice are resistant to HSV‐1 virus infection. Indeed, *Otulin* knockout increased the HSV‐1‐induced production of IFNβ and TNFα and finally decreased HSV‐1 viral replication (Figure 7G–I). This was consistent with increased levels of p‐IRF3, p‐TBK1 and p‐p65 in lungs of *Otulin^loxP/loxP^*‐*Lyz‐Cre* mice infected with HSV‐1 virus and reinforced linear ubiquitination of STING in the lung and liver of *Otulin^loxP/loxP^*‐*Lyz‐Cre* mice during DNA virus infection (Figure 7J–L), suggesting that OTULIN inhibits antiviral immune responses against DNA virus in vivo.

## 3 Discussion

The cGAS‐STING signaling is responsible for the immune surveillance of invading pathogens, especially DNA virus, and the dysregulation of this pathway would result in uncontrolled infections or various immunological disorders.^[^ ^55^ ^]^ Therefore, the duration and amplitude of the cGAS‐STING pathway must be stringently regulated. However, the exact molecular mechanisms controlling the initiation and termination of cGAS‐STING signaling remain obscure. In this study, we provide a previously unrecognized bidirectional regulation paradigm for HOIP and OTULIN‐governed STING linear ubiquitination in antiviral innate immunity (Figure S9D, Supporting Information). We find that HOIP binds to STING at the early stage of HSV‐1 infection, while the interaction between OTULIN and STING occurs at the late stage of HSV‐1 infection. HOIP deficiency attenuates antiviral immune responses against DNA virus, while OTULIN knockout boosts DNA virus‐triggered immune responses, suggesting that HOIP and OTULIN successively interact with STING to coordinately regulate cGAS‐STING pathway during different stages of DNA virus infection.

The regulation of the cGAS‐STING pathway is highly dependent on the organelle location and intracellular trafficking of certain key innate immune signaling molecules.^[^ ^56^ ^]^ As the central adaptor, STING activity is fine‐tuned by precise movement among multiple organelles.^[^ ^57^ ^]^ Proper vesicle trafficking and subcellular localization of STING are crucial for its normal physiological function, whereas its aberrant vesicular trafficking would lead to pathological tissue injury.^[^ ^36^, ^58^ ^]^ Previous studies have reported that the translocation of STING from the ER to the Golgi apparatus is mainly dependent on the COPII complex,^[^ ^6^, ^9^ ^]^ a multisubunit complex composed of secretion‐associated Ras‐related GTPase 1 (Sar1) and the heterodimers of Sec23/Sec24 and Sec13/Sec31.^[^ ^59^ ^]^ Among them, the Sec24 subunit serves as the primary site for cargo recognition through direct or indirect interacting with cargo proteins.^[^ ^60^ ^]^ Here, we find that DNA virus infection triggers the binding of the linear ubiquitinated STING to the Sec24b subunit of the COPII complex, which further facilitates STING trafficking from the ER to the Golgi apparatus. Previous study has reported that Sec24c, another subunit of the COPII complex, is essential for STING trafficking and signaling,^[^ ^6^ ^]^ our results showed that HOIP deficiency didn't hinder STING's interaction with Sec24c, indicating that LUBAC‐mediated linear ubiquitination is not required for Sec24c‐dependent STING trafficking. Therefore, STING could translocate from the ER to the Golgi apparatus through linear ubiquitin‐dependent and ‐independent pathways. Our findings unravel a previously unknown molecular mechanism by which LUBAC‐mediated linear ubiquitination of STING promotes COPII‐dependent STING trafficking to the Golgi apparatus, thus triggering effective antiviral innate immune responses against DNA virus infection.

As a newly‐identified type of post‐translational modification, linear ubiquitination has been implicated in VSV and influenza virus infections through targeting RIG‐I‐MAVS or Janus kinase‐signal transducer and activator of transcription (JAK‐STAT) signaling pathways.^[^ ^34^, ^61^, ^62^, ^63^ ^]^ However, the physiological functions and the detailed regulatory mechanisms of linear ubiquitination in DNA virus infection remain unknown. In this study, we reveal that STING, the key component in cGAS‐STING signaling, is a physiological substrate of linear ubiquitination, which could be reversibly modified by LUBAC and OTULIN during different stages of DNA virus infection. Although previous studies have reported that STING could be ubiquitinated by different E3 ligases, such as RING‐finger protein 5 (RNF5), tripartite motif‐56 (TRIM56) and mitochondrial E3 ubiquitin protein ligase 1 (MUL1), to regulate STING protein stability, dimerization and recruitment of downstream signaling molecules,^[^ ^37^ ^]^ it remains unclear whether and how ubiquitination regulates STING transport from the ER to the Golgi apparatus. Here, we found that LUBAC could catalyze the linear ubiquitination of STING at the early stage of DNA virus infection and promote STING trafficking from the ER to the Golgi apparatus through binding to the Sec24b subunit of COPII complex, thereby facilitating the effective activation of antiviral innate immune responses against DNA virus. Various linear ubiquitin chain‐binding proteins, such as NEMO and ABIN‐1, have been reported to be key regulators in NF‐κB‐mediated signaling.^[^ ^64^ ^]^ Sec24b, like NEMO and ABIN‐1, also binds to the linear ubiquitin chains via a distinct zinc finger‐like domain. We further confirmed that the deletion of the zinc finger‐like domain of Sec24b abolishes its interaction with STING, indicating that Sec24b promotes the ER‐to‐Golgi translocation of STING by functioning as a reader protein of linear ubiquitination. These findings expand our understanding on the assembly, recognition and removal of linear ubiquitination in antiviral innate immune responses against DNA virus

Linear ubiquitination plays a critical role in NF‐κB signaling pathway and a recent work showed that STING induces HOIP‐mediated synthesis of linear ubiquitin chains to stimulate NF‐κB signaling.^[^ ^65^, ^66^ ^]^ We did find that STING recruited HOIP and promoted NF‐κB activation. We further showed that STING lysine 338 was ubiquitinated by LUBAC and mutation of STING (K338R) decreased TBK1 phosphorylation and subsequent NF‐κB activation, providing a further regulatory mechanism for STING activation. Appropriately controlled STING‐mediated immune responses are beneficial for the host defense against invading pathogens, whereas excessive STING‐dependent inflammatory responses could lead to serious tissue damage and induce inflammatory‐related diseases, such as STING‐associated vasculopathy.^[^ ^67^, ^68^ ^]^ Here, we reveal a linear ubiquitination‐governed spatiotemporal regulatory mechanism that fine‐tunes STING‐driven antiviral immunity, which provides a potential therapeutic strategy against infectious diseases via targeting STING.

In summary, our findings reveal a dynamic spatiotemporal regulatory mechanism of cGAS‐STING signaling pathway governed by linear ubiquitination. We identify STING as a physiological substrate of linear ubiquitination, which could be successively and reversibly regulated by LUBAC and OTULIN through mutually exclusive interactions during different phases of DNA virus infection. LUBAC conjugates the linear ubiquitin chains on STING to facilitate Sec24b‐dependent vesicle trafficking of STING from the ER to the Golgi apparatus, whereas OTULIN is recruited by phosphorylated STING to remove the linear ubiquitin chains on STING, thus promoting lysosome‐mediated STING degradation to prevent excessive antiviral innate immune responses. Our study provides mechanistic insights into the orchestrated spatiotemporal regulation of cGAS‐STING signaling pathway by linear ubiquitination, which suggests potential drug targets for various STING‐associated infectious and immunological diseases.

## 4 Experimental Section

### Mice and Cells

*Hoip^loxP/loxP^* mice were obtained from RIKEN^[^ ^30^ ^]^ and *Otulin^loxP/loxP^* mice were generated by GemPharmatech Co., Ltd. In detail, *Otulin^loxP/loxP^* mice were generated using a gene‐targeting strategy in ES cells in which the target cassette was composed of exon 2 of the *Otulin* gene flanked by loxP sites. *Lyz‐Cre* mice were from the Jackson Laboratory. *Hoip^loxP/loxP^* mice or *Otulin^loxP/loxP^* mice were crossed with *Lyz‐Cre* mice to obtain myeloid‐cell specific *Hoip*‐deficient or *Otulin*‐deficient (*Hoip^loxP/loxP^*‐*Lyz‐Cre* or *Otulin^loxP/loxP^*‐*Lyz‐Cre*) mice. For animal experiments, eight to ten weeks‐old *Hoip^loxP/loxP^*‐*Lyz‐Cre* or *Otulin^loxP/loxP^*‐*Lyz‐Cre* and littermate controls with normal *Hoip* or *Otulin* expression (*Hoip^loxP/loxP^* or *Otulin^loxP/loxP^*) were used. All mice were maintained in specific pathogen‐free (SPF) facility and all animal experiments were carried out under the supervision of the Animal Care and Use Committee of the Institute of Microbiology (SQIMCAS2022099), Chinese Academy of Sciences, Beijing, China.

### Cell lines

Human THP‐1 was maintained in RPMI‐1640 medium (Cytiva/HyClone, SH30809.01) with 10% fetal bovine serum (FBS, Gibco, 10091148). Human HEK293T, Vero, and iBMDM cells were cultured in Dulbecco's modified Eagle's medium (DMEM, Gibco, C11995500BT) with 10% FBS. BMDMs were isolated and cultured as previously described.^[^ ^69^ ^]^ All cells were grown at 37 °C in a 5% CO_2_ humidified incubator. The shHOIP and shOTULIN THP‐1 cells were established using lentiviral shRNA delivery strategies.^[^ ^30^ ^]^ Lentiviral particles were produced in HEK293T cells by transient cotransfection of lentiviral packaging plasmids, and lentiviral vectors expressing shRNA against HOIP or OTULIN using Lipofectamine 2000 (Invitrogen, 11668019). Forty‐eight hours later, the lentiviral supernatant was used to infect THP‐1 cells along with 8 µg/mL polybrene (Santa Cruz, sc‐134220). Cells were selected with 2 µg/mL puromycin and the levels of HOIP or OTULIN protein expression were measured using immunoblot analysis. iBMDM cells were kindly provided by F. Shao (National Institute of Biological Sciences, Beijing, China), which were generated by using J2 retrovirus.^[^ ^70^ ^]^ *Sting* ^−/‐^ and *Sec24b* ^−/‐^ iBMDMs were generated using CRISPR‐Cas9 system. iBMDMs were transfected with PX458 vectors containing sgRNA targeting STING using Lipofectamine 2000 (Lipo2000). GFP‐positive cells were sorted by flow cytometry using the BD FACSAria III cell sorter (BD Biosciences) and confirmed by immunoblot analysis. WT STING and STING (K338R) re‐expressed stably *Sting* ^−/−^ iBMDM were constructed by transfected Flag‐STING and Flag‐STING (K338R) using Lipo2000. WT OTULIN and OTULIN (C129A) re‐expressed stably *Otulin* ^−/−^ iBMDM were constructed by transfected Flag‐OTULIN and Flag‐OTULIN (C129A) using Lipo2000. Two days after transfection, 550 µg/mL G418 (Sigma–Aldrich, A1720) was added to the growth media to select for WT STING, STING (K338R), WT OTULIN, and OTULIN (C129A) expressing stably cells. WT Sec24b and Sec24b (ΔZF) re‐expressed stably *Sec24b* ^−/−^ iBMDM were constructed by transfected Myc‐Sec24b and Myc‐Sec24b (ΔZF) using Lipo2000. WT HOIP and HOIP (C885S) re‐expressed stably *Hoip* ^−/−^ iBMDM were constructed by transfected Myc‐HOIP and Myc‐HOIP (C885S) using Lipo2000. Two days after transfection, 2 µg/mL blasticidin (Invitrogen, A1113903) was added to the growth media to select for WT Sec24b, Sec24b (ΔZF), WT HOIP and HOIP (C885S) expressing stably cells.

### Plasmids, Reagents, and Viruses

HA‐cGAS and Myc‐STING were gifts from Chengjiang Gao (Shandong University). RIG‐I, MAVS, TBK1, and IRF3 were provided by Xin Ye (Institute of Microbiology, Chinese Academy of Sciences). All the plasmids of STING, HOIP, OTULIN, and their mutants were sequenced at the Beijing Genomics Institute (BGI) for verification. VSV and SeV virus were gifts from Xin Ye (Institute of Microbiology, Chinese Academy of Sciences). The HSV‐1 virus was a gift from George Fu Gao (Institute of Microbiology, Chinese Academy of Sciences) and the VACV virus was a gift from Pengyan Xia (Peking University). Compound 11a was reported previously^[^ ^35^ ^]^ and synthesized from WuXi AppTec Co., Ltd. The reagents were purchased from the following companies: Poly(I:C)‐HMW/LyoVecTM (Invivogen, tlrl‐piclv), ISD (Invivogen, tlrl‐isdc), cGAMP (Invivogen, tlrl‐nacga23), Biotin‐cGAMP (BioLog Life Science institute, C157) and BX‐795 (Selleck, S1274). For preparation of virus, viruses were incubated with Vero cells, followed by supernatant collection 48 h later. Supernatants were ultra‐centrifuged at 25 000 × *g* for 2 h Pellets were resuspended in RPMI‐1640 medium.

### Viral Infection and Plaque Assay

THP‐1 or other cells were plated 24 h before infection. Cells were infected with virus for indicated times. Viral titers were determined in Vero cells using a standard plaque assay. For mice infection, 8–10 weeks‐old and sex matched mice of different genotypes were infected with HSV‐1 (1 × 10^6^ plaque forming units (PFU) per mouse) via tail vein injection and the survival of the infected mice was monitored for 10 days. The sera were collected for Enzyme‐linked immunosorbent assay (ELISA) to measure the productions of IFN‐β and TNF‐α. The viral titers in brains, liver, and spleen were determined by standard plaque assays. Lung from infected mice were fixed and stained with hematoxylin and eosin solution for histological analysis. All animal studies were approved by the Biomedical Research Ethics Committee of Institute of Microbiology, Chinese Academy of Sciences (SQIMCAS2022099).

### Quantitative Real‐Time PCR and Enzyme‐Linked Immunosorbent Assay

Quantitative real‐time PCR (qRT‐PCR) and ELISA were performed as previously described.^[^ ^71^ ^]^ Briefly, total cellular RNAs were isolated with FastPure Cell/Tissue Total RNA Isolation Kit (Vazyme, RC101‐01), according to the manufacturer's instructions. qRT‐PCR analysis was carried out using the ABI 7300 Detection System (Applied Biosystems) and Hieff qPCR SYBR Green Master Mix (YEASEN, 11203ES03). Specific primers used for qRT‐PCR assays are shown in Table S1, Supporting Information. ELISA kits including mouse IFN‐β ELISA kit (Mbbiology, MB‐6317A) and mouse TNF‐α ELISA kit (Raybiotech, ELM‐TNFa) for IFN‐β and TNF‐α were used to quantify the respective cytokines according to manufacturer's instructions.

### Immunoprecipitation and Immunoblot Analysis

For immunoprecipitation (IP), whole‐cell extracts were lysed in the Western and IP buffer (Beyotime, P0013J), and supernatants were collected and incubated with anti‐Flag M2 Affinity Gel. After 4 h of incubation, beads were washed five times with lysis buffer. The immunoprecipitates or whole‐cell lysates were loaded and subjected to SDS‐PAGE and then blotted with specific antibodies. For immunoblot analysis, cells were lysed with Western and IP buffer or RIPA buffer (Beyotime, P0013B) supplemented with a protease inhibitor cocktail (MedChemExpress, HY‐K0010). Protein concentrations were measured with bicinchoninic acid protein assay kit (Beyotime, P0010), and made equal in different samples with cell lysis buffer. Proteins were separated by SDS‐PAGE gel and then transferred onto polyvinylidene difluoride membranes (Millipore, IPVH00010). After incubation with primary and secondary antibodies, blots were subsequently developed using Immobilon Western Chemiluminescent HRP Substrate (Millipore, WBKLS0500) before being exposed to X‐ray film. The following antibodies were used: anti‐phospho‐STING antibody (Cell Signaling Technologies, 19781S, 1:1000), anti‐STING antibody (Abcam, ab239074, 1:1,000), anti‐phospho‐IRF3 antibody (Cell Signaling Technologies, 29047S, 1:1,000), anti‐IRF3 antibody (Cell Signaling Technologies, 4302, 1:1,000), anti‐phospho‐TBK1 antibody (Cell Signaling Technologies, 5483, 1:1,000), anti‐TBK1 antibody (Cell Signaling Technologies, 3504, 1:1,000), anti‐phospho‐p65 antibody (Cell Signaling Technologies, 3031S, 1:1,000), anti‐p65 antibody (Cell Signaling Technologies, 8242S, 1:1,000), anti‐Tubulin antibody (Sigma–Aldrich, T6199, 1:1,000), anti‐β‐actin antibody (Sigma–Aldrich, A2228, 1:1,000), anti‐HOIP antibody (Invitrogen, PA5‐61812, 1:1,000), anti‐SHARPIN antibody (Proteintech, 14626‐1‐AP, 1:1,000), anti‐Myc antibody (Cell Signaling Technologies, 2276S, 1:1,000), anti‐Flag antibody (Sigma–Aldrich, F1804, 1:1,000), anti‐HA antibody (Abcam, ab236632, 1:1,000), anti‐linear Ub antibody (LifeSensors, AB130, 1:500), anti‐Sec24a antibody (Thermofisher, PA5‐100110, 1:1,000), anti‐Sec24b antibody (Thermofisher, A304‐876A, 1:1,000), anti‐HSV‐1‐ICP8 antibody (Santa Cruz, sc‐53330, 1:1,000), anti‐IRhom2 antibody (Thermofisher, PA5‐48602, 1:1,000), anti‐His antibody (ABclonal, AE003, 1:1,000), anti‐GST antibody (ABclonal, AE006, 1:1,000), anti‐GM130 antibody (Abcam, ab52649, 1:1,000), anti‐HOIL‐1L antibody (Sigma–Aldrich, HPA024185, 1:1,000), anti‐OTULIN antibody (Invitrogen, PA5‐25638, 1:1,000), anti‐GAPDH antibody (YTHX biotech, ZB002, 1:5,000), anti‐Ub antibody (Invitrogen, 13–1600, 1:1,000), anti‐ubiquitin specific for K63 antibody (Cell Signaling Technologies, 5621S, 1:1,000), anti‐ubiquitin specific for K48 antibody (Cell Signaling Technologies, 8081S, 1:1,000), anti‐Sec24c antibody (Abcam, ab241336, 1:1,000), anti‐Sec13 antibody (ABclonal, A11613, 1:1,000), anti‐Sec31a antibody (ProteinTech, 17913‐1‐AP, 1:1,000), anti‐AP1Sl antibody (Thermofisher, A305‐396A, 1:1,000), anti‐HGS antibody (ABclonal, A1790, 1:1,000), anti‐VPS37a antibody (ABclonal, A7853, 1:1,000), anti‐Calnexin antibody (Cell Signaling Technologies, 2679, 1:200), anti‐GCC2 antibody (ABclonal, A13814, 1:1000), anti‐ISG15 antibody (Proteintech, 15981‐1‐AP, 1:1,000), anti‐SUMO antibody (Cell Signaling Technologies, 4971S, 1:1,000), Goat‐anti‐Rabbit Alexa Fluor 488 (ZSGB‐BIO, ZF‐0511, 1:200), Goat‐anti‐Rabbit Alexa Fluor 594 (ZSGB‐BIO, ZF‐0516, 1:200), Goat‐anti‐Mouse HRP (ZSGB‐BIO, ZB‐2305, 1:10,000), Goat‐anti‐Rabbit HRP (ZSGB‐BIO, ZB‐2306, 1:10,000).

### Ubiquitination Assay

Ubiquitylation assays were described in the previous studies.^[^ ^72^, ^73^ ^]^ Cells transfected with indicated plasmids were pelleted with 1% SDS and boiled in 100 °C for 10 min to destroy the interactions, followed by dilution in TNE buffer (50 mM Tris (pH 7.5), 150 mM NaCl, 1% NP‐40, 1% Triton X‐100, 0.5% sodium deoxycholate, 10 mM NaF, 1 mM Na3VO4 and proteinase inhibitor (Selleck)) with 0.1% SDS for subsequent ubiquitinated substrates immunoprecipitation and immunoblotting.

### In vitro Deubiquitinase Assay

In vitro deubiquitination assays were described in the previous studies.^[^ ^74^ ^]^ In brief, for purification of linear ubiquitinated STING, 20 cm^2^ control cells transfected with Flag‐tagged STING, Myc‐tagged HOIP, HA‐tagged HOIL1L were lysed for SDS boiling. Subsequent immunoprecipitation of SDS‐boiling lysates was performed using anti‐Flag antibody (Medical & Biological Laboratories Co., Ltd.) and protein A/G agarose beads (Santa Cruz) in TNE buffer at 4 °C, followed with the elution in 0.1M glycine, pH2.7. Flag peptides were then concentrated with 0.5ml 30 KD centrifugal filters (Millipore) to 100 µL. STING purified in HOIP knock‐down cells was a negative control without linear ubiquitination. For in vitro deubiquitination assays, 10 µL linear ubiquitinated STING incubated with 4 µg His‐OTULIN‐wildtype (or inactive mutant C129A) in a total 40 µL DUB reaction buffer (50 mM Tris (pH 7.5), 50 mM NaCl and 5 mM DTT) at 37 °C for 90 min and then immunoblotted with indicated antibodies.

### Native PAGE for STING Oligomers

Cells were lysed in native lysis buffer (50 mM Tris‐HCl, pH 7.5, 150 mM NaCl, 1% NP‐40, 10% glycerol, and protease inhibitor cocktails). Cells then were disrupted using super‐sonication in ice water. Lysates were centrifuged at 12 000 g for 5 min to achieve the supernatant. The supernatant was added to 5 × Protein Loading Buffer (Yeason, 20317ES05) and subjected to native PAGE (Coolaber, SK6011‐50T) in cold room. Immunoblotting was performed with antibodies against STING (Abcam, ab239074).

### Immunofluorescence Assay

iBMDM Cells were cultured on 24‐mm diameter cover glass placed in 6‐well culture plate. After HSV‐1 infection, cells were fixed in 4% paraformaldehyde, permeabilized with 0.2% Triton X‐100, and blocked with 1% BSA for 1 h. Cells were stained with indicated antibodies, followed by counterstaining with DAPI. Confocal images were taken with the Leica SP8 confocal microscope (Leica Microsystems) and analyzed by the Leica Application Suite Las X (v2.0.1.14392) software. Relative fluorescence intensities were measured using Image J along the arrows. The quantitated colocalization was determined by fluorescence intensities. About 100 cells were counted and analyzed for each biological replicate.

### in vitro Pull‐Down Assay

For the precipitation assay, Glutathione Sepharose 4B GST‐tagged protein purification resin (Cytiva, 17075605, 20 µl) was coated with 10 µg GST fusion protein or GST control in binding buffer (50 mM Tris, pH 7.5, 150 mM NaCl, 5 mM DTT and 0.1% NP‐40) supplemented with 1% protease inhibitor cocktail for 2 h at 4 °C, and incubated with specified amounts of soluble recombinant protein. After 2 h of incubation at 4 °C, beads were extensively washed and bound proteins were subjected to SDS‐PAGE.

### GST‐UBAN Pull Down Assay

For isolation of linear Ub chains modified proteins, recombinant protein containing four UBAN region from human NEMO (residues 257–346) fused to GST was used.^[^ ^45^ ^]^ Cells were lysed in HEPES lysis buffer (20 mM HEPES, PH 7.2, 150 mM NaCl, 0.5% Triton X‐100, 1 mM NaF and 1 mM dithiothreitol). GST‐tagged UBAN were purified from bacteria BL21 and bound to Glutathione sepharose 4B beads, followed by incubation in cell lysates at 4 °C overnight. Linear ubiquitinated STING bound to GST‐UBAN‐beads were washed for three times by using HEPES lysis buffer, and followed by immunoblot analysis.

### Mass Spectrum (MS) Analysis of STING Ubiquitination Sites

Control or HOIP knockdown HEK293T cells transiently transfected with plasmids encoding Flag‐STING with or without HSV infection, were lysed with buffer (20 mM Na_2_HPO_4_, 20 mM NaH_2_PO_4_, 1% NP‐40, 2 mM EDTA) supplemented with 1 mM DTT, 5 mM NEM, complete protease inhibitor cocktail, PhosSTOP (Roche, 04906837001) and 0.1% SDS. The lysates were incubated with anti‐Flag M2 Affinity Gel (Sigma–Aldrich, A2220). The bound material was eluted with 0.1 M glycine‐HCl pH 2.7, separated by electrophoresis in a 10% gel, and stained with Bio‐Safe Coomassie and then for mass spectrometry analysis.

Bands representing putative interacting proteins were cut out and subjected to in‐gel digestion with sequencing grade modified trypsin. The resulting peptide mixtures were analyzed by the LC‐MS/MS system that consisted of an online Easy‐nLC 1200 nano‐HPLC system (Thermo Fisher Scientific) and an Orbitrap Exploris 480 mass spectrometer with the FAIMS interface (Thermo Fisher Scientific). The mass spectrometer was operated in the positive‐ion mode at an ion transfer tube temperature of 320 °C. The positive‐ion spray voltage was 2.2 kV. For a full mass spectrometry survey scan, the scan ranged from 350 to 1500 m/z at a resolution of 60 000 and a maximum injection time of 50 ms. The MS2 spectra were acquired at a resolution of 15 000 with a normalized AGC target of 75% and a maximum injection time was set to 22 ms, and the dynamic exclusion duration was set to 45 s. The acquired MS/ MS spectra were searched against the human Uniprot (updated on 20220418; 20,376 protein entries) with Thermo Proteome Discoverer (2.4.1.15). The mass tolerances were 15 ppm for precursor ions and 0.05 Da for productions. Peptides and proteins were filtered with a false discovery rate of 1% using the decoy database strategy.

### Gel‐Filtration Chromatography Assays

Cells were lysed in a lysis buffer containing 50 mM Tris‐HCl, pH 7.5, 150mM NaCl, 1 mM MgCl_2_, 1 mM DTT, 10 mM NaF, 1 mM Na_3_VO_4,_ and a protease inhibitor cocktail (Complete EDTA‐free, Roche) and passed through 0.45 µm polycarbonate filters (PALL). The lysates were injected onto a Superdex 200 HR (10/30) column (GE Lifesciences, 28990944) and fractionated in PBS buffer pH 7.5 (137 mM NaCl, 2.7 mM KCl, 10 mM Na_2_HPO_4,_ and 2 mM KH_2_PO_4_) using an AKTA chromatography system (Amersham). One column volume of eluates was fractionated with 500 µL in each fraction, at the elution speed of 0.6 mL min^−1^. Aliquots (100 µL each) of each fraction were resolved by SDS‐PAGE gels and detected with indicated antibodies.

### Statistics

Data shown in graphs are expressed as the mean ± SD. Unpaired two‐tailed Student's *t*‐tests and two‐way ANOVA Sidak's multiple comparisons test and Tukey's multiple comparisons test were used for statistical analysis. *P < 0.05, **P < 0.01, ***P < 0.001 or ****P < 0.0001 were considered to be statistically significant.

## Conflict of Interest

The authors declare no conflict of interest.

## Author Contributions

Y.Z., Y.F., and L.Q. contributed equally to this work. C.H.L and L.Z conceived and designed the experiments. Y.Z, Y.F, C.H.L, and L.Z analyzed the data and wrote the manuscript. Y.Z., Y.F., and L.Q performed the majority of the experiments with the assistance of M.Z., Zhe. L., Z.Z., G.C., Zehui. L., Q.C., P.G., B.L., and J.W. All authors critically reviewed, discussed the results, contributed and agreed to the final version of the manuscript.

## Supporting information



Supporting Information

## Data Availability

The mass spectrometry proteomics data have been publicly released via the iProX partner repository with the dataset identifier PXD046415. Unprocessed gel images and all data presented in the graphs are available as source data files. Source data are provided with this paper. All other data supporting the findings of this study are available from the corresponding author on reasonable request.
