# Supplementary material for: Spatiotemporal Regulation of STING Activity by Linear Ubiquitination Governs Antiviral Immunity
Source: Adv Sci (Weinh). 2025 Jun 19;12(28):2417660. doi: 10.1002/advs.202417660 (PMC12302577; doi:10.1002/advs.202417660)
Supplement: Supplementary file 1 — Supporting Information [file ADVS-12-2417660-s001.docx]

**
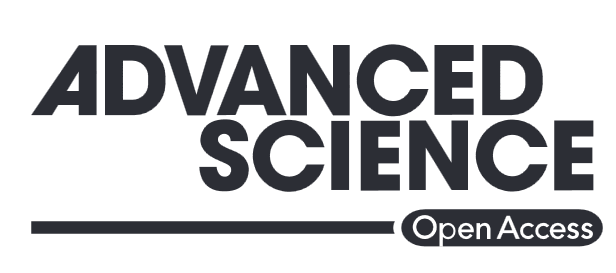
**

Supporting Information

Linear ubiquitination drives STING trafficking to govern

the kinetics of antiviral immunity

*Yong Zhang†, Yesheng Fu†, Lihua Qiang†, Mengyuan Zhao, Zhe Lu, Zhuo Zhao, Guoping Chen, Zehui Lei, Qiyao Chai, Pupu Ge, Bingxi Li, Jing Wang, Cui Hua Liu*, and Lingqiang Zhang**

**This PDF file includes:**

Figs. S1 to S9

Table S1

**Supporting Information**

**
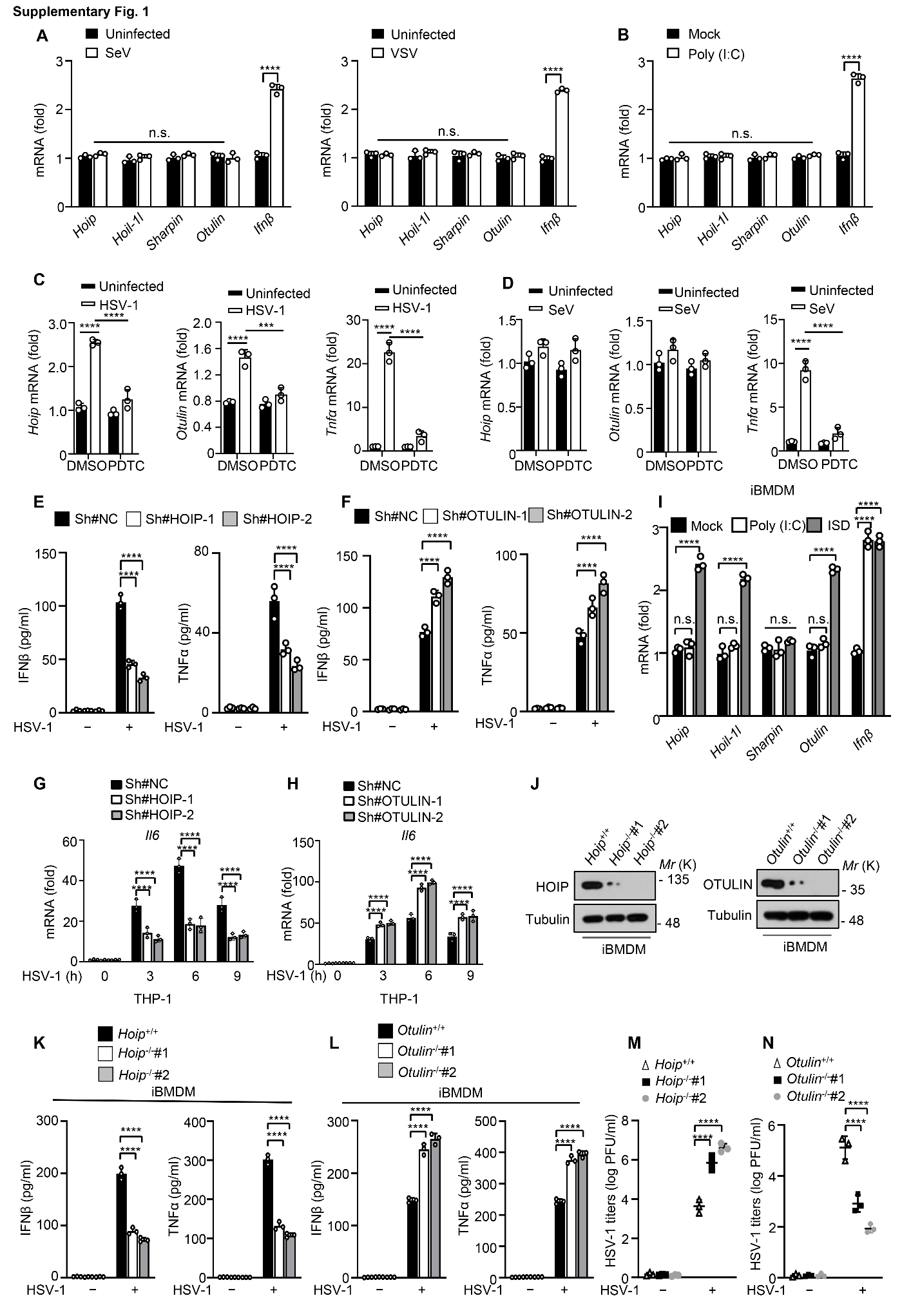
**

**Figure S1. HOIP promotes while OTULIN inhibits DNA virus-induced antiviral immune responses, related to Figure 1.** (**A**) qRT-PCR analysis of *Hoip*, *Hoil-1l*, *Sharpin*, *Otulin* or *Ifn-β* mRNA in THP-1 cells infected with SeV or VSV for 8 hours. (**B**) qRT-PCR analysis of *Hoip*, *Hoil-1l*, *Sharpin*, *Otulin* or *Ifn-β* mRNA in THP-1 cells treated with 5 μg/ml Poly (I: C) for 4 hours. (**C** and **D**) qRT-PCR analysis of *Hoip*, *Otulin* or *Tnfα* mRNA in THP-1 cells treated with HSV-1 (**C**) or SeV (**D**) for 6 hours. (**E**) ELISA analysis of IFNβ or TNFα in WT or *HOIP* knockdown THP-1 cells infected with HSV-1 for 8 hours. (**F**) ELISA analysis of IFNβ or TNFα in WT or *OTULIN* knockdown THP-1 cells infected with HSV-1 for 8 hours. (**G** and **H**) qRT-PCR analysis of *Il6* mRNA in WT, HOIP knockdown (**G**) or OTULIN knockdown (**H**) THP-1 cells infected with HSV-1 for the indicated time points. (**I**) qRT-PCR analysis of *Hoip*, *Hoil-1l*, *Sharpin*, *Otulin* or *Ifn-b* mRNA in iBMDM cells treated with 5 μg/ml Poly (I: C) or 2 μg/ml ISD for 4 hours. (**J**) Immunoblot analysis of iBMDM cells transduced with HOIP sgRNA or OTULIN sgRNA. (**K** and **L**) ELISA analysis of IFNβ and TNFα in WT, *Hoip* knockout (**K**) or *Otulin* knockout (**L**) iBMDM cells infected with HSV-1 for 8 hours. **(M** and **N)** Plaque assay of HSV-1 titers in WT, *Hoip* knockout (**M**) or *Otulin* knockout (**N**) iBMDM cells infected with HSV-1 for 8 hours. Data are presented as the mean ± SD. Statistical significance was determined by two-way ANOVA with sidak’s multiple comparisons test (**A** and **B**) or with Tukey’s multiple comparisons test (**C**, **D**, **E**, **F**, **G**, **H**, **I**, **K, L**, **M** and **N**). ****P* < 0.001, *****P* < 0.0001; n.s., not significant. Data are representative of three independent experiments.

**
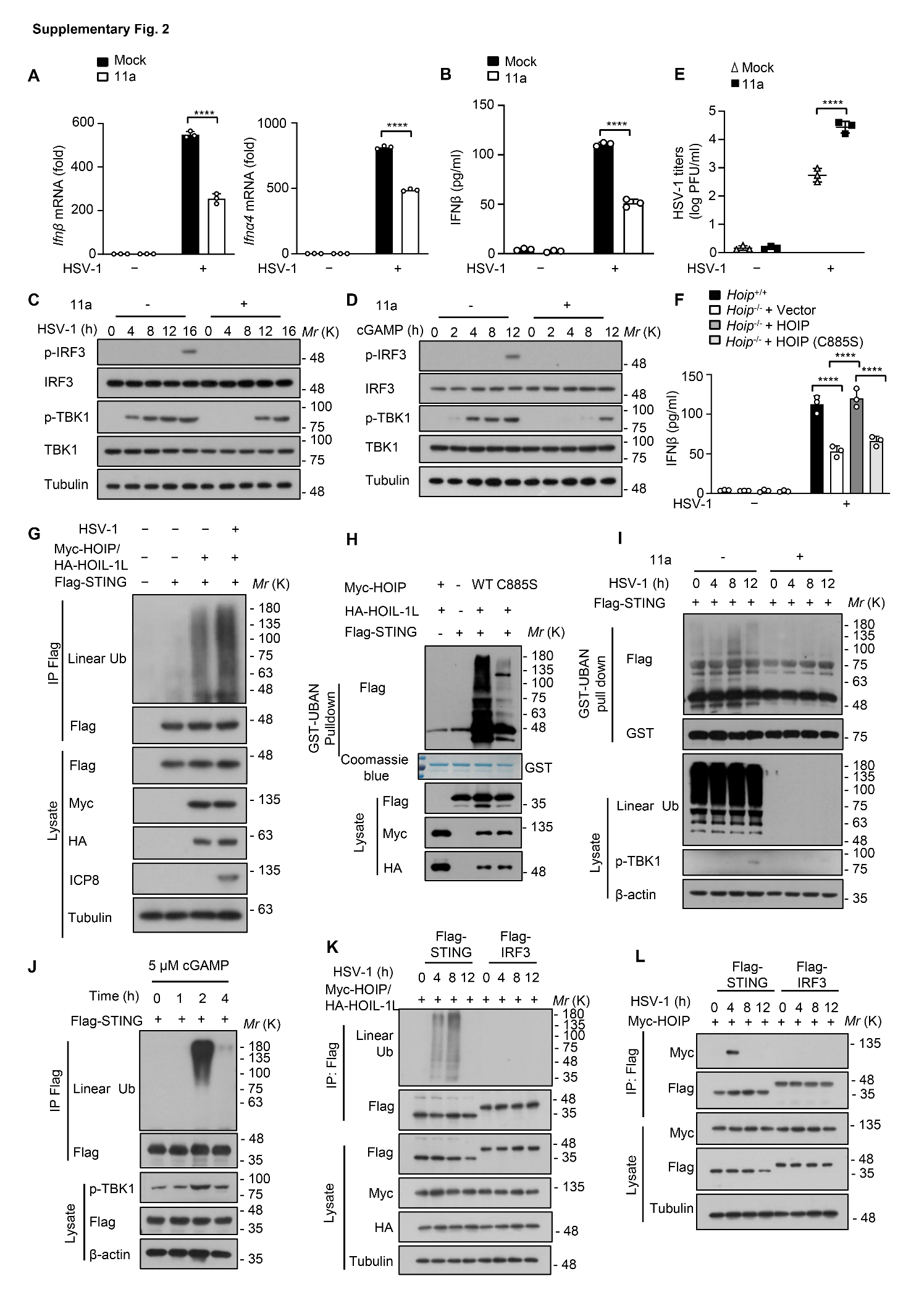
Figure S2. The E3 ubiquitin ligase activity of LUBAC is required for DNA virus-induced antiviral immune responses, related to Figure 2.** (**A**) qRT-PCR analysis of *IFN-β* and *IFN-α4* in THP-1 cells pretreated with 20 μM 11a for 12 hours, followed by HSV-1 virus infection for 6 hours. (**B**) ELISA analysis of IFN-β in THP-1 cells pretreated with 20 μM 11a for 12 hours, followed by HSV-1 virus infection for 6 hours. (**C**) Immunoblot analysis of p-IRF3, IRF3, p-TBK1, TBK1 and Tubulin in HEK293T cells pretreated with 20 μM 11a for 12 hours, followed by HSV-1 virus infection for the indicated times. (**D**) Immunoblot analysis of p-IRF3, IRF3, p-TBK1, TBK1 and Tubulin in HEK293T cells pretreated with 20 μM 11a for 12 hours, followed by treatment with 5 μM cGAMP for the indicated time points. (**E**) Plaque assay of HSV-1 titers in THP-1 cells infected with HSV-1 virus for 6 hours in the absence or presence of 20 μM 11a. (**F**) ELISA analysis of IFNβ in *Hoip*^-/-^ iBMDM cells complemented with WT HOIP or HOIP (C885S) mutant infected with HSV-1 for 6 hours. (**G**) Immunoblot analysis of the linear polyubiquitination of STING in HEK293T cells transfected with indicated plasmids using Lipo2000, followed by infected with or without HSV-1 for 6 hours. (**H**) Immunoblot analysis of the linear ubiquitination of STING in HEK293T cells transfected with Flag-STING, HA-HOIL-1L, Myc-HOIP or Myc-HOIP (C885S) using Lipo2000. GST-tagged UBAN used to isolate linear Ub chains were subjected to SDS-polyacrylamide gel for coomassie brilliant blue staining. (**I**) Immunoblot analysis of the linear ubiquitination of STING in the absence or presence of 20 μM 11a, followed by infected with HSV-1 for the indicated time points. (**J**) Immunoblot analysis of the linear ubiquitination of STING in HEK293T cells transfected with Flag-STING using Lipo2000, followed by treatment with 5 μM cGAMP for the indicated time points. (**K**) Immunoblot analysis of the linear ubiquitination of STING (or IRF3) in HEK293T cells transfected with Flag-STING (or Flag-IRF3) using Lipo2000, followed by treatment with HSV-1 for the indicated time points. (**L**) Immunoprecipitation analysis of the interaction between STING (or IRF3) and HOIP in HEK293T cells cotransfected with Flag-STING (or Flag-IRF3) and Myc-HOIP using Lipo2000, followed by treatment with HSV-1 for the indicated time points. Data are presented as the mean ± SD. Statistical significance was determined by two-way ANOVA with sidak’s multiple comparisons test (**A**, **B** and **E**) and with Tukey’s multiple comparisons test (**F**). *****P* < 0.0001. Data are representative of three independent experiments.

**
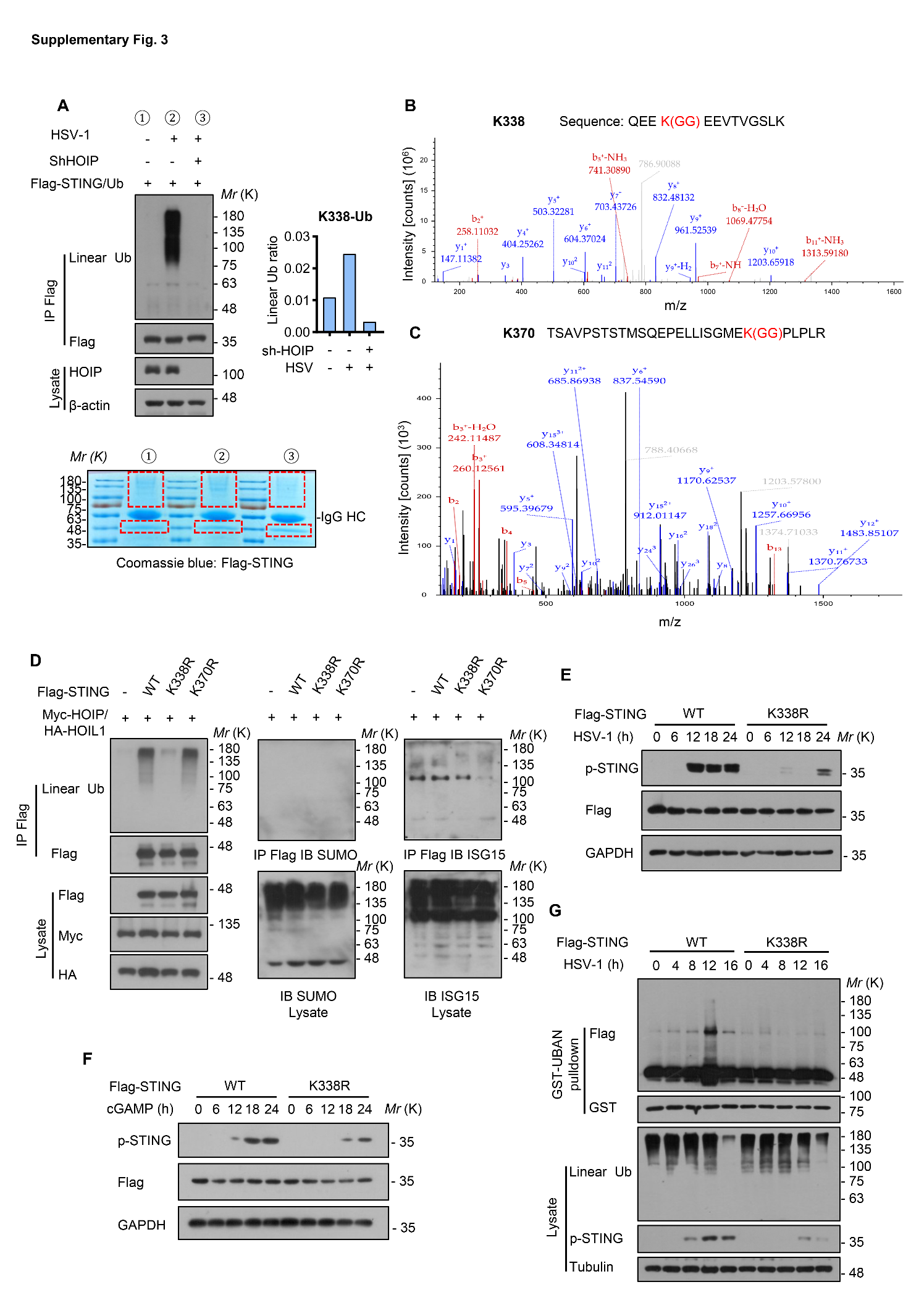
Figure S3. Lysine 338 of STING is the major site modified by LUBAC, related to Figure 3.** (**A**) Coomassie Brilliant Blue staining and immunoblot analysis of Flag-STING from HEK293T cells in the absence or presence of ShHOIP, followed by infected with HSV-1 for 6 hours. The right graph indicates the relative MS intensity of the K338-modified peptides of STING (normalized to the amount of STING) that detected in all samples from (**A**). (**B**) LC-MS spectra of the polyubiquitination of STING at Lys 338. (**C**) LC-MS spectra of the polyubiquitination of STING at Lys 370. (**D**) Immunoblot analysis of the linear polyubiquitination, sumoylation and ISGylation of STING in HEK293T cells cotransfected with Flag-STING, Flag-STING (K338R) or Flag-STING (K370R) using Lipo2000. (**E**) Immunoblot analysis of p-STING, Flag-STING or GAPDH in HEK293T cells transfected with Flag-STING or Flag-STING (K338R) mutant using Lipo2000, followed by infected with HSV-1 for the indicated time points. (**F**) Immunoblot analysis of p-STING, Flag-STING or GAPDH in HEK293T cells transfected with Flag-STING or Flag-STING (K338R) mutant using Lipo2000, followed by treatment with 2 μg/ml cGAMP for the indicated time points. (**G**) Immunoblot analysis of linear ubiquitination of STING in HEK293T cells expressing WT STING or STING (K338R) mutant, followed by infected with HSV-1 for the indicated time points. Data are representative of three independent experiments.

**
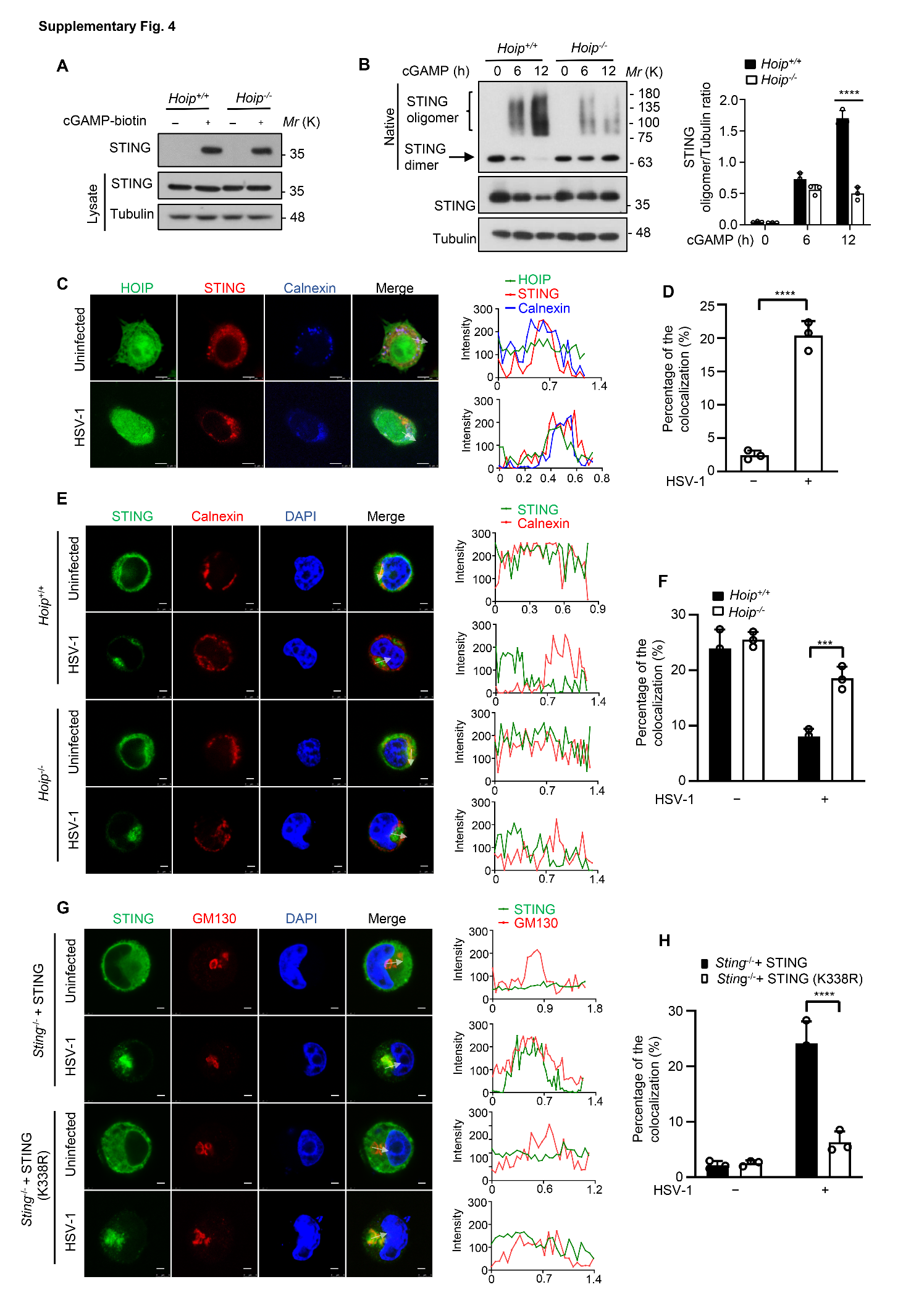
Figure S4. The mutation of STING K338R blocks the trafficking of STING from ER to Golgi apparatus, related to Figure 4.** (**A**) Immunoblot analysis of the interaction of STING and transfected biotin-cGAMP using Lipo2000 in *Hoip*^+/+^ and *Hoip*^-/-^ iBMDM cells, followed by immunoprecipitation with streptavidin magnetic beads and immunoblot analysis with anti-STING antibody. (**B**) Immunoblot analysis of oligomeric and dimeric STING in *Hoip*^+/+^ and *Hoip*^-/-^ iBMDM cells infected with HSV-1 for the indicated time points. The densitometry quantitative analysis of STING oligomer relative to Tubulin is shown besides. (**C**) Immunofluorescence analysis of the colocalization of endogenous STING, HOIP and calnexin in iBMDM cells, followed by infected with HSV-1 for 6 hours. Scale bars, 10 μm. Relative fluorescence intensities of STING, HOIP and calnexin were measured using Image J along the arrows. (**D**) The percentage of colocalization of STING, HOIP and calnexin. The quantitated colocalization was determined by fluorescence intensities. About 100 cells were counted and analyzed for each biological replicate. (**E**) Immunofluorescence analysis of the colocalization of endogenous STING and calnexin in *Hoip*^+/+^ and *Hoip*^-/-^ iBMDM cells, followed by infected with HSV-1 for 6 hours. Scale bars, 10 μm. Relative fluorescence intensities of STING and calnexin were measured using Image J along the arrows. (**F**) The percentage of colocalization of STING with calnexin. The quantitated colocalization was determined by fluorescence intensities. About 100 cells were counted and analyzed for each biological replicate. (**G**) Immunofluorescence analysis of the colocalization of STING and GM130 in *Sting*^-/-^ and *Sting*^-/-^ iBMDM cells complemented with STING or STING (K338R) infected with HSV-1 for 6 hours. Scale bars, 10 μm. Relative fluorescence intensities of STING and GM130 were measured using Image J along the arrows. (**H**) The percentage of colocalization of STING or STING (K338R) with Golgi marker GM130. The quantitated colocalization was determined by fluorescence intensities. About 100 cells were counted and analyzed for each biological replicate. Data are presented as the mean ± SD. Statistical significance was determined by two-way ANOVA with sidak’s multiple comparisons test (**B, F** and **H**) and unpaired two-tailed Student’s t-tests (**D**). ****P* < 0.001, *****P* < 0.0001. Data are representative of three independent experiments.


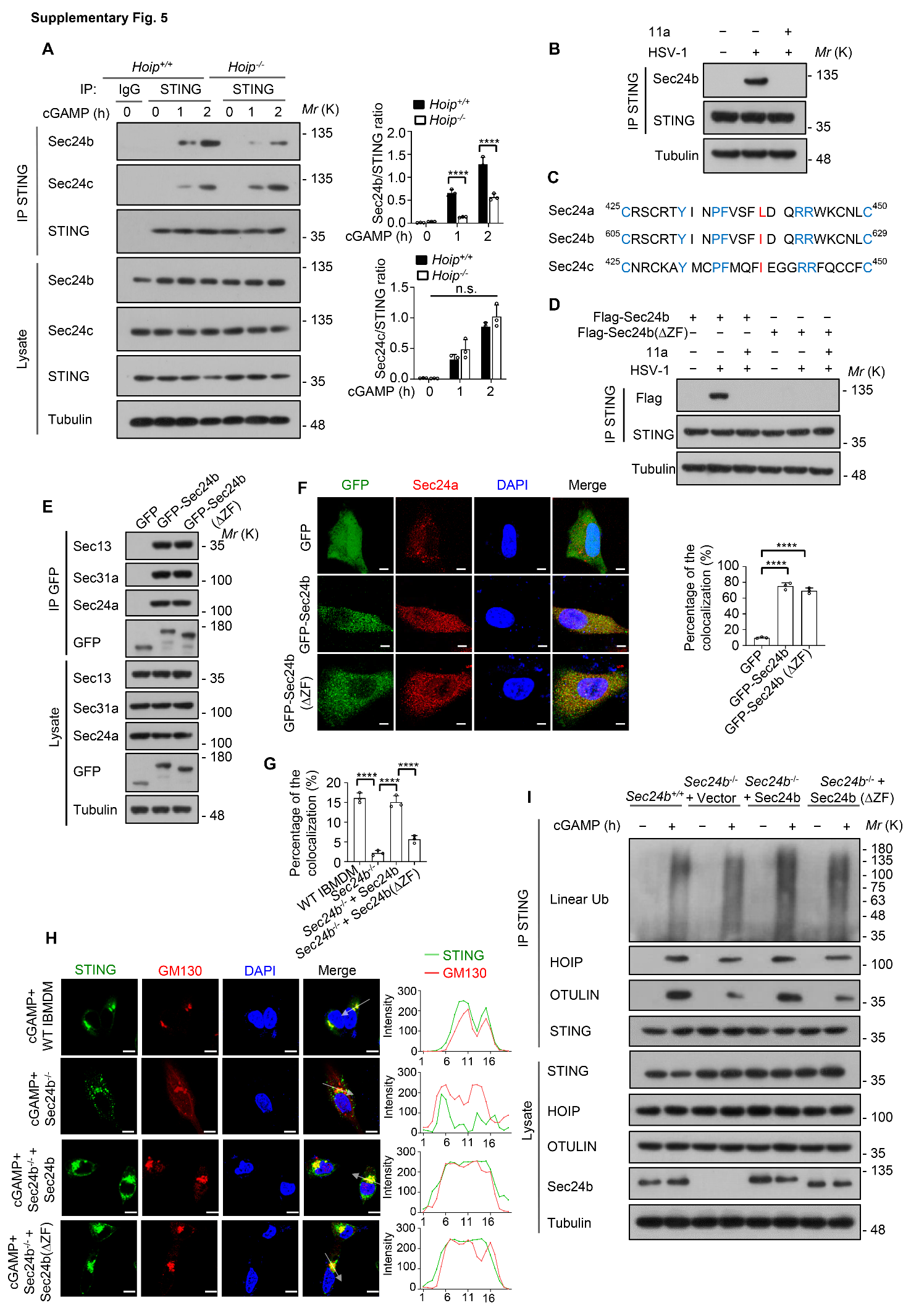


**Figure S5. The Sec24b ZF domain is required for STING trafficking from the ER to the Golgi apparatus, related to Figure 4.** (**A**) Immunoblot analysis of the interaction between endogenous STING and Sec24b or Sec24c in *Hoip*^+/+^ and *Hoip*^-/-^ iBMDM cells treated with 2 μg/ml cGAMP for the indicated time points. The densitometry quantitative analysis of Sec24b or Sec24c relative to STING are shown on the right. (**B**) Immunoblot analysis of the interaction between endogenous STING and Sec24b in the absence or presence of 20 μM 11a, followed by infected with HSV-1 for 6 hours. (**C**) A schematic diagram of Sec24s. (**D**) Immunoblot analysis of the interaction between transfected Flag-Sec24b, Flag-Sec24b (∆ZF) and endogenous STING using Lipo2000 in the absence or presence of 20 μM 11a, followed by infected with HSV-1 for 6 hours. (**E**) Immunoblot analysis of the interaction between GFP, GFP-Sec24b or GFP-Sec24b (ΔZF) and Sec13, Sec31a or sec24a in 293T cells. (**F**) Immunofluorescence analysis of the colocalization of Sec24a and GFP, GFP-Sec24b or GFP-Sec24b (ΔZF) in A549 cells. Scale bars, 10 μm. The percentage of colocalization of GFP with Sec24a on the right. About 100 cells were counted and analyzed for each biological replicate. (**G**) The percentage of colocalization of STING with Golgi marker GM130. About 100 cells were counted and analyzed for each biological replicate. (**H**) Immunofluorescence analysis of the colocalization of endogenous STING and GM130 in *Sec24*^+/+^ or *Sec24b*^-/-^ iBMDM cells complemented with Sec24b or Sec24b (∆ZF), followed by infected with HSV-1 for 6 hours. Scale bars, 10 μm. Relative fluorescence intensities of STING and GM130 were measured using Image J along the arrows. The quantitated colocalization was determined by fluorescence intensities. (**I**) Immunoblot analysis of the linear ubiquitination of STING and the interactions between endogenous STING and HOIP or OTULIN in *Sec24*^+/+^ or *Sec24b*^-/-^ iBMDM cells complemented with Sec24b or Sec24b (∆ZF), followed by treatment with 2 μg/ml cGAMP for 2 hours. Data are presented as the mean ± SD. Statistical significance was determined by two-way ANOVA with sidak’s multiple comparisons test (**A** and **G**) and ordinary one-way comparisons test (**F**). *****P* < 0.0001; n.s., not significant. Data are representative of three independent experiments.

**
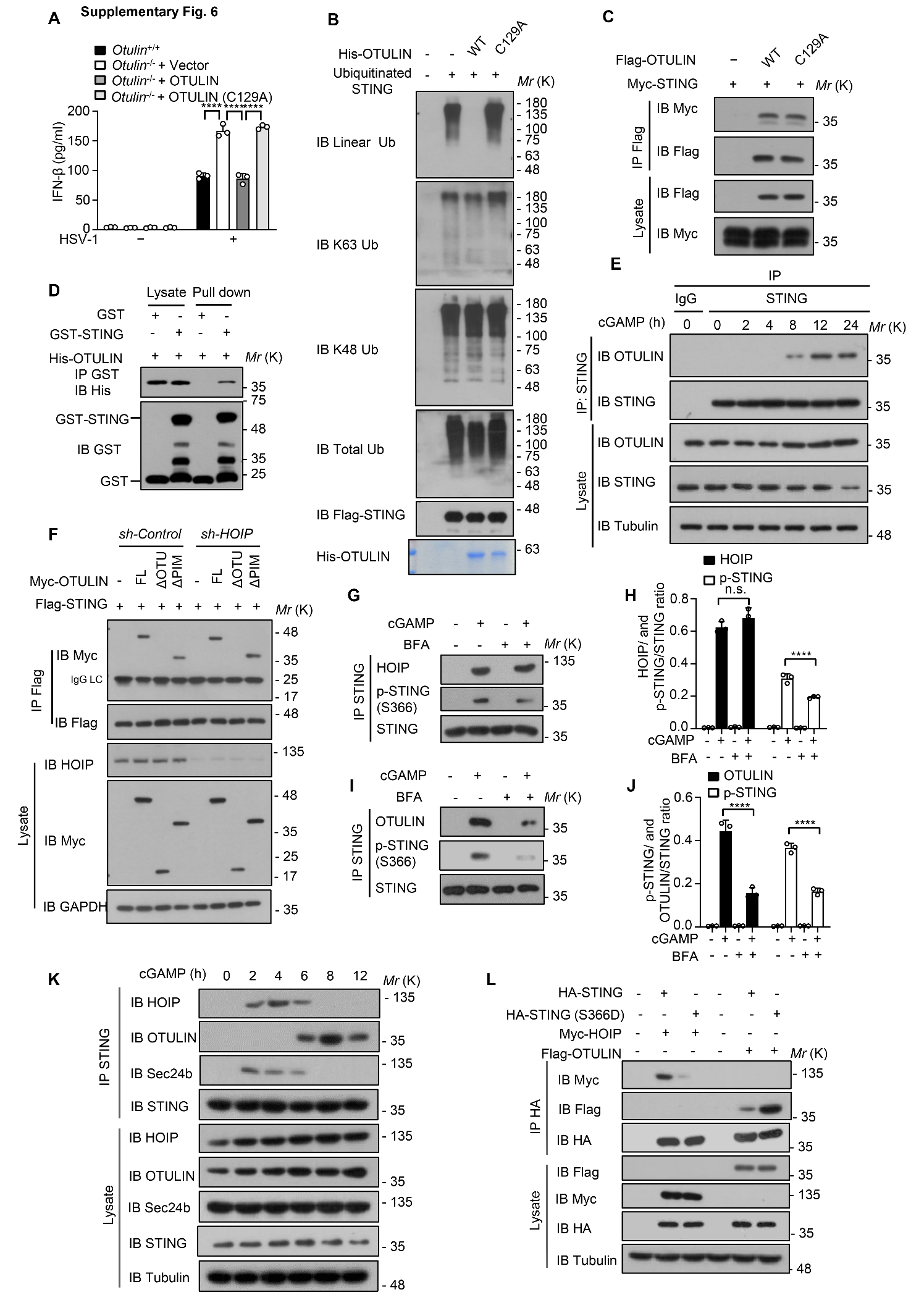
**

**Figure S6. OTULIN interacts with STING at the late stage of HSV-1 infection and removes the linear ubiquitination of STING, related to Figure 5.** (**A**) ELISA analysis of IFN-β in *Otulin*^-/-^ iBMDM cells complemented with OTULIN or OTULIN (C129A) mutant infected with HSV-1 for 6 hours. (**B**) Immunoblot analysis of *in vitro* deubiquitinating assay of STING by purified His-OTULIN or His-OTULIN (C129A). His-OTULIN or His-OTULIN (C129A) were purified from *E.coli* BL21 and were subjected to SDS-polyacrylamide gel for coomassie brilliant blue staining. (**C**) Immunoblot analysis of the interaction between Flag-OTULIN, Flag-OTULIN (C129A) and Myc-STING. (**D**) Immunoblot analysis of GST-pull down between GST-STING and His-OTULIN. (**E**) Immunoblot analysis of the interactions between endogenous OTULIN and STING in iBMDM cells treated with 2 μg/ml cGAMP for the indicated time points. (**F**) Immunoprecipitation analysis of the interaction between OTULIN mutants and STING in HEK293T cells transfected with sh-Control or sh-HOIP using Lipo2000. (**G, H**) Immunoblot analysis of the p-STING and the interaction between endogenous STING and HOIP in THP-1 cells stimulated with 2 μg/ml cGAMP for 4 hours in the absence or presence of 20 μM Brefeldin A (BFA) (**G**). The densitometry quantitative analysis of HOIP or p-STING relative to STING is shown on the right (**H**). (**I, J**) Immunoblot analysis of the p-STING and the interaction between endogenous STING and OTULIN in THP-1 cells stimulated with 2 μg/ml cGAMP for 8 hours in the absence or presence of 20 μM Brefeldin A (BFA) (**I**). The densitometry quantitative analysis of OTULIN or p-STING relative to STING is shown on the right (**J**). (**K**) Immunoblot analysis of the interactions between STING and HOIP, OTULIN or Sec24b in BMDM cells treated by 2 μg/ml cGAMP for the indicated time points. (**L**) Immunoblot analysis of the interactions between STING (S366D) and HOIP/OTULIN in HEK293T cells transfected with HA-STING, HA-STING (S366D), Myc-HOIP or Flag-OTULIN. Data are presented as the mean ± SD. Statistical significance was determined by two-way ANOVA with sidak’s multiple comparisons test (**A**, **H**, **J**). *****P* < 0.0001; n.s., not significant. Data are representative of three independent experiments.

**
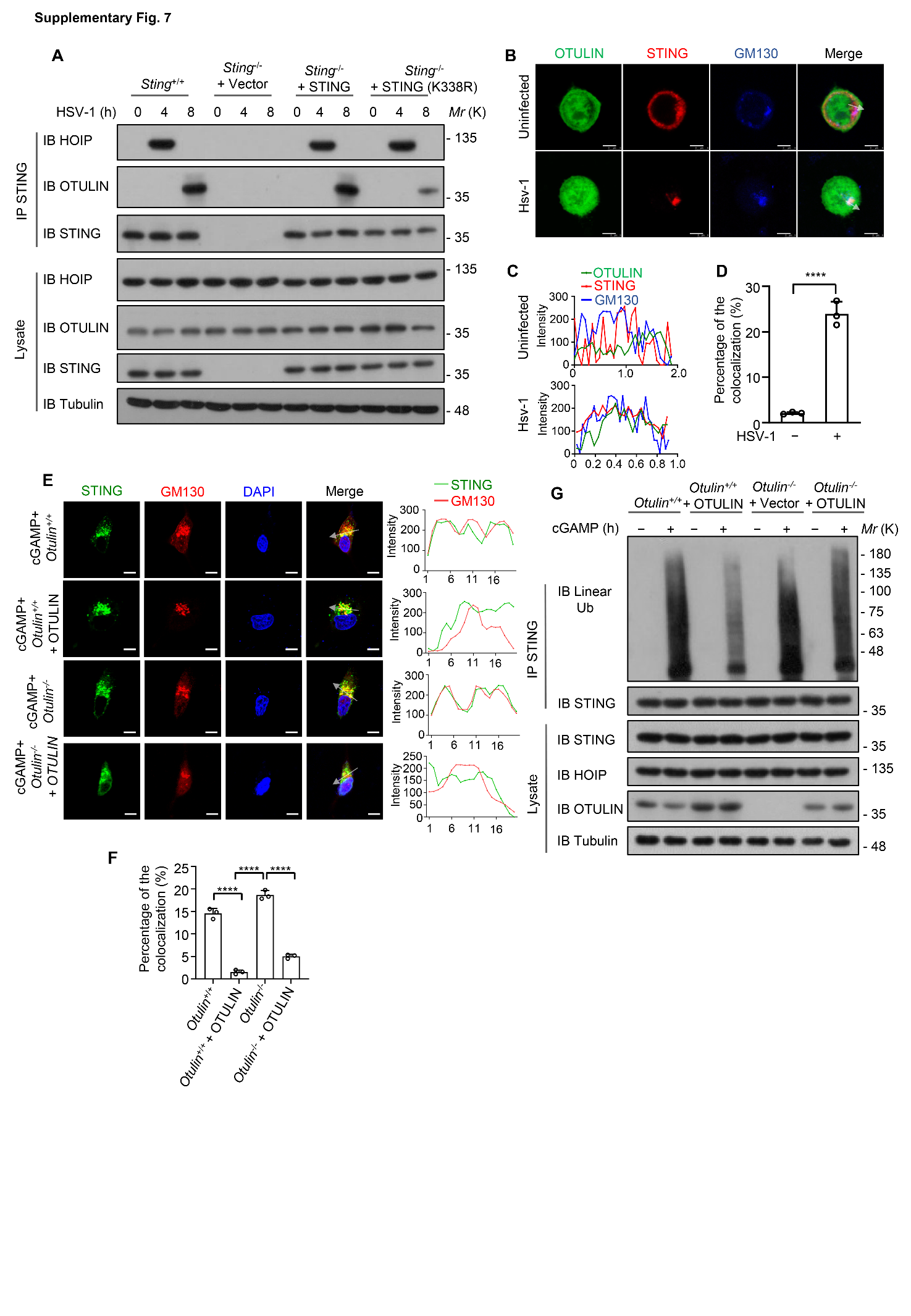
**

**Figure S7. Overexpression or knockout of OTULIN regulates the STING trafficking and the linear ubiquitination of STING, related to Figure 5.** (**A**) Immunoblot analysis of the interaction between endogenous STING and HOIP or OTULIN in *Sting*^-/-^ iBMDM cells complemented with STING or STING (K338R) mutant infected with HSV-1 for the indicated time points. (**B**) Immunofluorescence analysis of the colocalization of endogenous STING, OTULIN and GM130 in iBMDM cells, followed by infected with HSV-1 for 6 hours. Scale bars, 10 μm. (**C**) Relative fluorescence intensities of STING , OTULIN and GM130 were measured using Image J along the arrows. (**D**) The percentage of colocalization of STING, OTULIN and GM130. The quantitated colocalization was determined by fluorescence intensities. About 100 cells were counted and analyzed for each biological replicate. (**E**) Immunofluorescence analysis of the colocalization of endogenous STING and GM130 in *Otulin*^+/+^, *Otulin*^+/+^ expressing OTULIN or *Otulin*^-/-^ iBMDM cells expressing vector or OTULIN, followed by treatment with 2 μg/ml cGAMP for 4 hours. Scale bars, 10 μm. Relative fluorescence intensities of STING and GM130 were measured using Image J along the arrows in the middle. The quantitated colocalization was determined by fluorescence intensities. (**F**) The percentage of colocalization of STING with Golgi marker GM130 is shown. About 100 cells were counted and analyzed for each biological replicate. (**G**) Immunoblot analysis of the linear ubiquitination of STING and the interactions between endogenous STING and HOIP or OTULIN in *Otulin*^+/+^, *Otulin*^+/+^ expressing OTULIN or *Otulin*^-/-^ iBMDM cells expressing vector or OTULIN, followed by treatment with 2 μg/ml cGAMP for 4 hours. Data are presented as the mean ± SD. Statistical significance was determined by unpaired two-tailed Student’s t-tests (**D**) and ordinary one-way comparisons test (**F**). *****P* < 0.0001; n.s., not significant. Data are representative of three independent experiments.

**
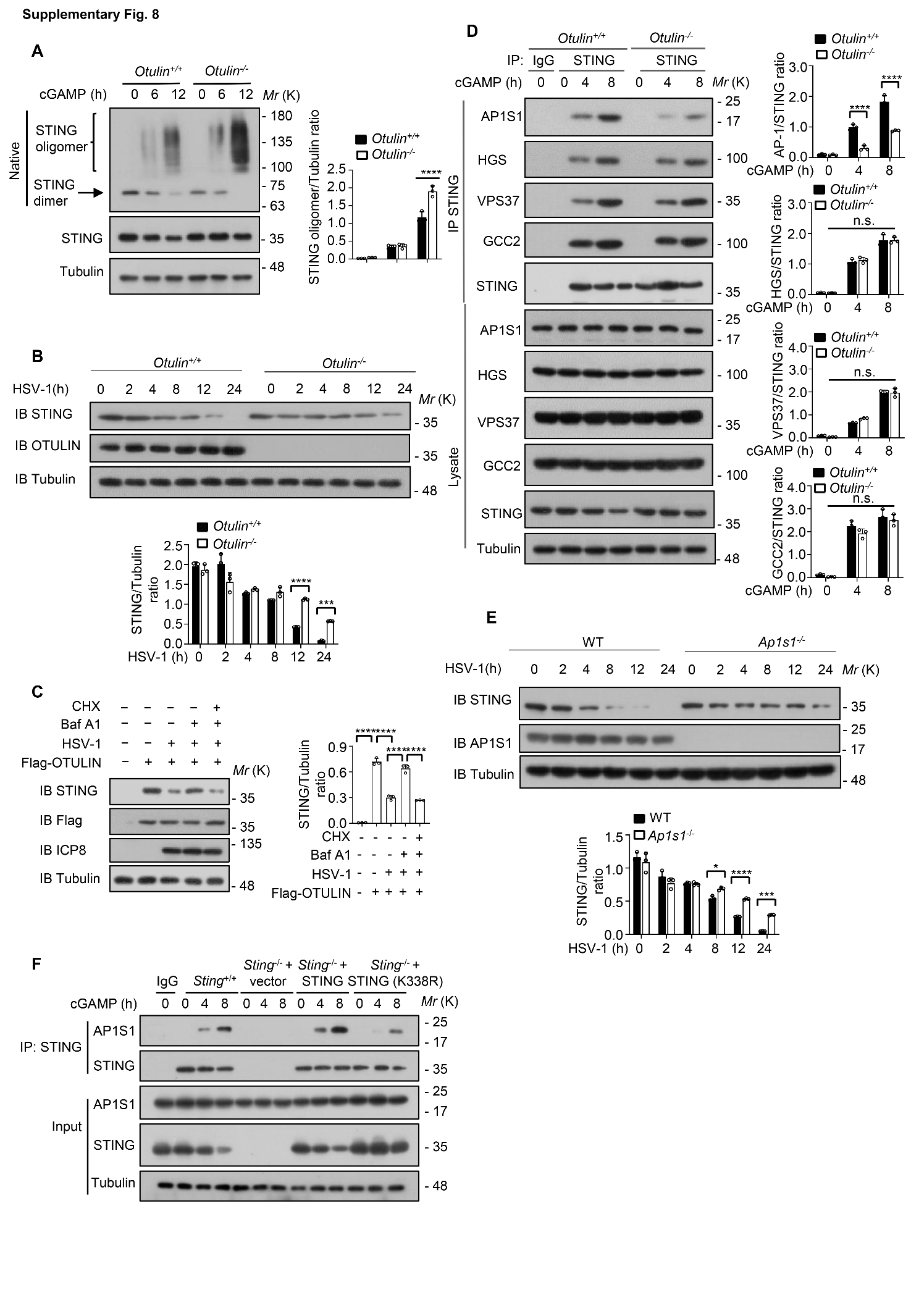
**

**Figure S8. The deubiquitinase OTULIN promotes STING degradation depending on AP-1, related to Figure 5.** (**A**) Immunoblot analysis of oligomeric and dimeric STING in *Otulin*^+/+^ and *Otulin*^-/-^ iBMDM cells stimulated with 2 μg/ml cGAMP for the indicated time points. The densitometry quantitative analysis of STING oligomer relative to Tubulin is shown on the right. (**B**) Immunoblot analysis of STING protein in *Otulin*^+/+^ or *Otulin*^-/-^ iBMDM infected with HSV-1 for indicated time points. The densitometry quantitative analysis of STING, relative to Tubulin is shown at the bottom. (**C**) Immunoblot analysis of STING protein in HEK293T cells treated with 0.2 μM Baf A1 and 10 μM CHX after infection with HSV-1 for 12 hours. The densitometry quantitative analysis of STING, relative to Tubulin is shown on the right. (**D**) Immunoblot analysis of the interaction between endogenous STING and AP1S1, HGS, VPS37, or GCC2 in *Otulin*^+/+^ and *Otulin*^-/-^ iBMDM cells stimulated with 2 μg/ml cGAMP for the indicated time points. The densitometry quantitative analysis of AP1S1, HGS, VPS37, or GCC2 relative to STING is shown on the right. (**E**) Immunoblot analysis of STING protein in WT or *Ap1s1*^-/-^ iBMDM infected with HSV-1 for indicated time points. The densitometry quantitative analysis of STING, relative to Tubulin is shown at the bottom. (**F**) Immunoblot analysis of the interaction between endogenous STING and AP1S1 in *Sting^-/-^* iBMDM cells stably expressing WT STING or STING (K338R) infected with HSV-1 for indicated time points. Data are presented as the mean ± SD. Statistical significance was determined by ordinary one-way comparisons test (**C**) and two-way ANOVA with sidak’s multiple comparisons test (**A**, **B**, **D**, **E**). **P* < 0.05; ****P* < 0.001; *****P* < 0.0001; n.s., not significant. Data are representative of three independent experiments.

**
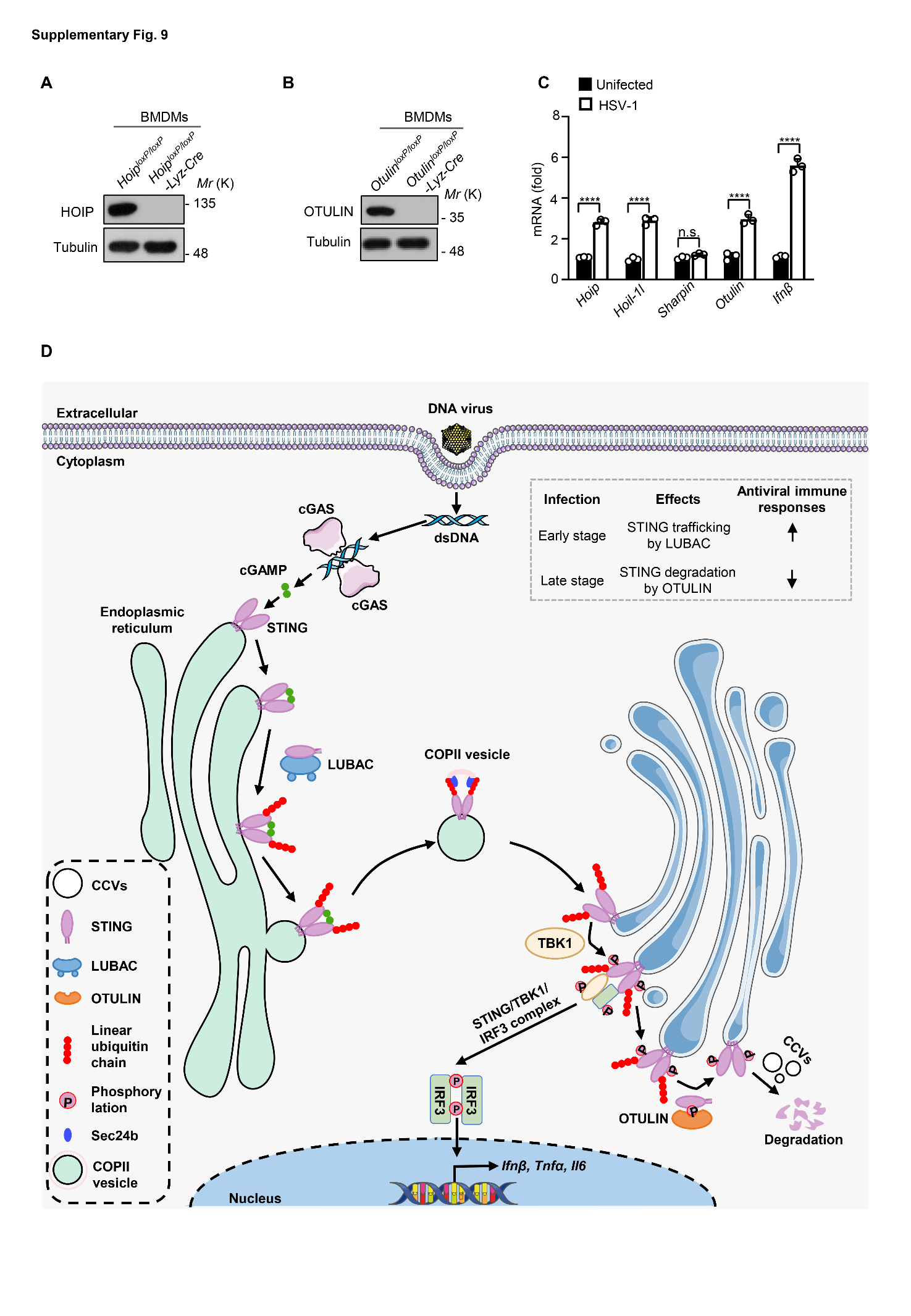
**

**Figure S9. HOIP promotes while OTULIN inhibits antiviral innate immune responses against DNA virus *in vivo*, related to Figure 6 & 7.** (**A, B**) Immunoblot analysis of the knockout efficiency of HOIP (**A**) or OTULIN (**B**) in BMDM cells. (**C**) qRT-PCR analysis of *Hoip*, *Hoil-1l*, *Sharpin*, *Otulin* or *Ifn-b* mRNA in BMDM cells infected with HSV-1 for 8 hours. (**D**) Proposed working model depicting the spatiotemporal regulation of STING signaling by linear ubiquitination-associated enzymes LUBAC and OTULIN during DNA virus infection. In response to DNA virus infection, LUBAC and OTULIN bidirectionally regulate immune responses against DNA viruses via targeting STING. During the early stage of DNA virus infection, LUBAC promotes linear ubiquitination of STING at lysine 338 to drive its trafficking depending on the Sec24b subunit of COPⅡ complex. At the late stage of DNA virus infection, OTULIN binds to phosphorylated STING and removes its linear ubiquitin chains to facilitate lysosome-mediated STING degradation, thus preventing excessive antiviral immune responses.

**Table S1. Plasmids and primers used in this study.**

| **Name** | **Description** | **Reference** |
| --- | --- | --- |
| **Plasmids** | | |
| pcDNA6A | T7 promoter, for mammalian expression, Myc tag, Amp^R^ | Invitrogen |
| pcDNA6A-HOIP | For expression of Myc-HOIP in mammalian cells | This study |
| pcDNA6A-HOIP-ZF | For expression of Myc-HOIP ZF domain in mammalian cells | This study |
| pcDNA6A-HOIP-UBA | For expression of Myc-HOIP UBA domain in mammalian cells | This study |
| pcDNA6A-HOIP-RBR | For expression of Myc-HOIP RBR domain in mammalian cells | This study |
| pcDNA6A-HOIP-LDD | For expression of Myc-HOIP LDD domain in mammalian cells | This study |
| pcDNA6A-HOIP (C885S) | For expression of Myc-HOIP (C885S) in mammalian cells | This study |
| pcDNA6A-Sec24a | For expression of Myc-Sec24a in mammalian cells | This study |
| pcDNA6A-Sec24b | For expression of Myc-Sec24b in mammalian cells | This study |
| pcDNA6A-IRhom2 | For expression of Myc-IRhom2 in mammalian cells | This study |
| pcDNA6A-OTULIN | For expression of Myc-OTULIN in mammalian cells | This study |
| pcDNA6A-OTULIN-∆PIM | For expression of Myc-OTULIN ∆PIM in mammalian cells | This study |
| pcDNA6A-OTULIN-∆OTU | For expression of Myc-OTULIN ∆OTU in mammalian cells | This study |
| pcDNA6A-STING | For expression of Myc-STING in mammalian cells | This study |
| pET30a | T7 promoter, for bacterial expression, 6xHis tag, Kan^R^ | Novagen |
| pET30a-OTULIN | For expression of recombinant protein His_6_-OTULIN | This study |
| pET30a-OTULIN (C129A) | For expression of recombinant protein His_6_-OTULIN (C129A) | This study |
| pET30a-HOIP-RBR-LDD | For expression of recombinant protein His_6_-HOIP RBR-LDD | This study |
| pET30a-Sec24b | For expression of recombinant protein His_6_-Sec24b | This study |
| pET30a-Sec24b (ΔZF) | For expression of recombinant protein His_6_-Sec24b (ΔZF) | This study |
| pGEX-6P-1 | *tac* promoter, for bacterial expression, GST tag, Amp^R^ | GE |
| pGEX-6P-1-STING | For expression of recombinant protein GST-STING | This study |
| pCDNA3.1-3×HA | CMV promoter, for mammalian expression, HA tag, AmpR | This study |
| pCDNA3.1-3×HA-cGAS | For expression of HA-cGAS in mammalian cells | This study |
| pCDNA3.1-3×HA-HOIL-1L | For expression of HA-HOIL-1L in mammalian cells | This study |
| p3xFlag-CMV14 | CMV promoter, for mammalian expression, 3xFlag tag, AmpR | Sigma-Aldrich |
| p3xFlag-CMV14-cGAS | For expression of Flag-cGAS in mammalian cells | This study |
| p3xFlag-CMV14-STING | For expression of Flag-STING in mammalian cells | This study |
| p3xFlag-CMV14-TBK1 | For expression of Flag-TBK1 in mammalian cells | This study |
| p3xFlag-CMV14-IRF3 | For expression of Flag-IRF3 in mammalian cells | This study |
| p3xFlag-CMV14-RIG-I | For expression of Flag-RIG-I in mammalian cells | This study |
| p3xFlag-CMV14-MAVS | For expression of Flag-MAVS in mammalian cells | This study |
| p3xFlag-CMV14-NEMO | For expression of Flag-NEMO in mammalian cells | This study |
| p3xFlag-CMV14-STING (1-240) | For expression of Flag-STING (1-240) in mammalian cells | This study |
| p3xFlag-CMV14-STING (1-160) | For expression of Flag-STING (1-160) in mammalian cells | This study |
| p3xFlag-CMV14-STING (41-379) | For expression of Flag-STING (41-379) in mammalian cells | This study |
| p3xFlag-CMV14-STING (81-379) | For expression of Flag-STING (81-379) in mammalian cells | This study |
| p3xFlag-CMV14-STING (111-379) | For expression of Flag-STING (111-379) in mammalian cells | This study |
| p3xFlag-CMV14-STING (151-379) | For expression of Flag-STING (151-379) in mammalian cells | This study |
| p3xFlag-CMV14-STING (221-379) | For expression of Flag-STING (221-379) in mammalian cells | This study |
| p3xFlag-CMV14-STING (K0) | For expression of Flag-STING (K0) in mammalian cells | This study |
| p3xFlag-CMV14-STING (K20) | For expression of Flag-STING (K20) in mammalian cells | This study |
| p3xFlag-CMV14-STING (K137) | For expression of Flag-STING (K137) in mammalian cells | This study |
| p3xFlag-CMV14-STING (K150) | For expression of Flag-STING (K150) in mammalian cells | This study |
| p3xFlag-CMV14-STING (K224) | For expression of Flag-STING (K224) in mammalian cells | This study |
| p3xFlag-CMV14-STING (K236) | For expression of Flag-STING (K236) in mammalian cells | This study |
| p3xFlag-CMV14-STING (K289) | For expression of Flag-STING (K289) in mammalian cells | This study |
| p3xFlag-CMV14-STING (K338) | For expression of Flag-STING (K338) in mammalian cells | This study |
| p3xFlag-CMV14-STING (K347) | For expression of Flag-STING (K347) in mammalian cells | This study |
| p3xFlag-CMV14-STING (K370) | For expression of Flag-STING (K370) in mammalian cells | This study |
| p3xFlag-CMV14-STING (K150/224/338/347/370R) | For expression of Flag-STING (K150/224/338/347/370R) in mammalian cells | This study |
| p3xFlag-CMV14-OTULIN (C129A) | For expression of Flag-OTULIN (C129A) in mammalian cells | This study |
| p3xFlag-CMV14-STING (S366A) | For expression of Flag-STING (S366A) in mammalian cells | This study |
| p3xFlag-CMV14-STING (S366D) | For expression of Flag-STING (S366D) in mammalian cells | This study |
| p3xFlag-CMV14-HOIP | For expression of Flag-HOIP in mammalian cells | This study |
| pEGFP-N1 | CMV promoter, for mammalian expression, GFP tag, KanR | Clontech |
| pEGFP-N1-Sec24b | For expression of GFP-Sec24b in mammalian cells | This study |
| pEGFP-N1-Sec24b (ΔZF) | For expression of GFP-Sec24b (ΔZF) in mammalian cells | This study |
| **Oligonucleotides (5’-3’) for qRT-PCR** | | |
| Sh#HOIP-1 | ACAGGTTGAAATGTTCCTT | This study |
| Sh#HOIP-2 | AATGGAAACTTGGACTGAA | This study |
| Sh#OTULIN-1 | CTCCTTTGGCTTCATATAT | This study |
| Sh#OTULIN-2 | ATCCTGCAAGTGCTCATTT | This study |
| sgRNA-*Hoip*-1-F | CACCGTTCACTGAGCGCCAATACCG | This study |
| sgRNA-*Hoip*-1-R | AAACCGGTATTGGCGCTCAGTGAAC | This study |
| sgRNA-*Hoip*-2-F | CACCGGTTGCAGCGGACCAAGCGTC | This study |
| sgRNA-*Hoip*-2-R | AAACGACGCTTGGTCCGCTGCAACC | This study |
| sgRNA-*Otulin*-1-F | CACCGACTTCCATAAGGCGAGTCCG | This study |
| sgRNA-*Otulin*-1-R | AAACCGGACTCGCCTTATGGAAGTC | This study |
| sgRNA-*Otulin*-2-F | CACCGGTGCACAGTAGTTATCACCA | This study |
| sgRNA-*Otulin*-2-R | AAACTGGTGATAACTACTGTGCACC | This study |
| sgRNA-*Sting*-1-F | CACCGCAGTAGTCCAAGTTCGTGCG | This study |
| sgRNA-*Sting*-1-R | AAACCGCACGAACTTGGACTACTGC | This study |
| sgRNA-*Sting*-2-F | CACCGCACCTAGCCTCGCACGAACT | This study |
| sgRNA-*Sting*-2-R | AAACAGTTCGTGCGAGGCTAGGTGC | This study |
| sgRNA-*Ap1s1*-1-F | CACCGATGCTACTGTTCAGCCGGCA | This study |
| sgRNA-*Ap1s1*-1-R | AAACTGCCGGCTGAACAGTAGCATC | This study |
| sgRNA-*Ap1s1*-2-F | CACCGCCAAGAGCTCTACGTATCGG | This study |
| sgRNA-*Ap1s1*-2-R | AAACCCGATACGTAGAGCTCTTGGC | This study |
| sgRNA-*Ap1s1*-3-F | CACCGTATTTCTGCTGCGCCATCGA | This study |
| sgRNA*-Ap1s1*-3-R | AAACTCGATGGCGCAGCAGAAATAC | This study |
| sgRNA-*Sec24b*-1-F | CACCGCCAGTGTACGCTGGGCCGCT | This study |
| sgRNA-*Sec24b*-1-R | AAACAGCGGCCCAGCGTACACTGGC | This study |
| sgRNA-*Sec24b*-2-F | CACCGGGAGTCCCCGAATTTTGTGT | This study |
| sgRNA-*Sec24b*-2-R | AAACACACAAAATTCGGGGACTCCC | This study |
| sgRNA-*Sec24b*-3-F | CACCGAGGGTTGATGTACGTCCGAC | This study |
| sgRNA*-Sec24b*-3-R | AAACGTCGGACGTACATCAACCCTC | This study |
| h*GAPDH*-F | GGAGCGAGATCCCTCCAAAAT | This study |
| h*GAPDH*-R | GGCTGTTGTCATACTTCTCATGG | This study |
| h*HOIP*-F | GAGCCCCGAAACTACCTCAAC | This study |
| h*HOIP*-R | CTTGACACCACGCCAGTACC | This study |
| h*HOIL-1L*-F | TGCTCAGATGCACACCGTC | This study |
| h*HOIL-1L*-R | CAAGACTGGTGGGAAGCCATA | This study |
| h*SHARPIN*-F | CAAGAGCAACTCACCACCAG | This study |
| h*SHARPIN*-R | TCTCTCTCCGTCAAGTTTCCAG | This study |
| h*OTULIN*-F | GGGGCATCAGAACCGAGATTA | This study |
| h*OTULIN*-R | TCGCCGTATGGAGGTGAACT | This study |
| h*IFN-β*-F | ATGACCAACAAGTGTCTCCTCC | This study |
| h*IFN-β*-R | GGAATCCAAGCAAGTTGTAGCTC | This study |
| h*IFN-**α4*-F | ACCTGGTTCAACATGGAAATG | This study |
| h*IFN-α4*-R | ACCAAGCTTCTTCACACTGCT | This study |
| h*CXCL-10*-F | GTGGCATTCAAGGAGTACCTC | This study |
| h*CXCL-10*-R | TGATGGCCTTCGATTCTGGATT | This study |
| h*TNF*-F | CCTCTCTCTAATCAGCCCTCTG | This study |
| h*TNF*-R | GAGGACCTGGGAGTAGATGAG | This study |
| m*Gapdh*-F | AGGTCGGTGTGAACGGATTTG | This study |
| m*Gapdh*-R | TGTAGACCATGTAGTTGAGGTCA | This study |
| m*Hoip*-F | GCCCTGAGGTGGGATTCTG | This study |
| m*Hoip*-R | TTGAGGTAGTTTCGAGGCTCC | This study |
| m*Hoil-1l*-F | CTGCTATCAAGTATGCCACCTG | This study |
| m*Hoil-1l*-R | TGTGCATGTACGCATCCTCC | This study |
| m*Sharpin*-F | GCTTCCGGCTAGGACTGTTAG | This study |
| m*Sharpin*-R | CTCGTGTTGGTTAGGCCCAC | This study |
| m*Otulin*-F | GAGACACATCCAATGACCCTG | This study |
| m*Otulin*-R | AGCATAGGCAAGAAGGAACATC | This study |
| m*Ifn-β*-F | CAGCTCCAAGAAAGGACGAAC | This study |
| m*Ifn-β*-R | GGCAGTGTAACTCTTCTGCAT | This study |
| m*Tnf*-F | CAGGCGGTGCCTATGTCTC | This study |
| m*Tnf*-R | CGATCACCCCGAAGTTCAGTAG | This study |
| GST-UBAN-F | GGATCCATGGGCATGCAGCTGGAAGAT | This study |
| GST-UBAN-R | GCGGCCGCTTAATGGTGATGATGATGATGGCTGCGACTGGCTTTCAG | This study |
